# Survival outcomes in patients with relapsed/refractory or MRD-positive B-cell acute lymphoblastic leukemia treated with blinatumomab

**DOI:** 10.1177/20406207231201454

**Published:** 2023-10-09

**Authors:** Hagop M. Kantarjian, Aaron C. Logan, Faraz Zaman, Nicola Gökbuget, Ralf C. Bargou, Yi Zeng, Gerhard Zugmaier, Franco Locatelli

**Affiliations:** Department of Leukemia, MD Anderson Cancer Center, The University of Texas, 1515 Holcombe Blvd., Unit 428, Houston, TX 77030, USA; Helen Diller Family Comprehensive Cancer Center, University of California San Francisco, San Francisco, CA, USA; Amgen Inc., Thousand Oaks, CA, USA; University Hospital, Frankfurt, Germany; Comprehensive Cancer Center Mainfranken, Uniklinikum Würzburg, Würzburg, Germany; Amgen Inc., Thousand Oaks, CA, USA; Amgen Research GmbH, Munich, Germany; Department of Pediatric Hematology/Oncology and Cell and Gene Therapy, IRCCS Bambino Gesù Children’s Hospital, Catholic University of the Sacred Heart, Rome, Italy

**Keywords:** blinatumomab, overall survival, relapsed/refractory B-cell ALL, relapse-free survival

## Abstract

Blinatumomab has demonstrated significant efficacy in adult and pediatric patients with relapsed/refractory B-cell acute lymphoblastic leukemia (R/R B-cell ALL) and patients with measurable residual disease (MRD). This review aimed to compare median relapse-free survival (RFS) and median overall survival (OS) in adult and pediatric patients with R/R or MRD-positive B-cell ALL from pivotal studies [MT-103-211 and TOWER for adults with Philadelphia chromosome (Ph)-negative R/R B-cell ALL, ALCANTARA for adults with Ph-positive R/R B-cell ALL, MT-103-203 for adults with MRD-positive B-cell ALL, and MT-103-205 for pediatric patients with R/R B-cell ALL], with the median RFS and OS from retrospective analyses, country or ethnicity-specific studies, and studies based on real-world evidence (RWE) identified from a literature search. Adults with Ph-negative R/R B-cell ALL who received blinatumomab as first salvage demonstrated a numerically longer median OS compared with that in patients from pivotal studies (MT-103-211 and TOWER) without additional safety concerns. In pediatric patients with R/R B-cell ALL treated with blinatumomab, the median RFS and OS from retrospective analyses and country/ethnicity-specific studies were comparable with the median RFS and OS from the pivotal study MT-103-205. The median RFS and OS from RWE studies in adults with R/R B-cell ALL were numerically longer than the median RFS and OS from pivotal studies (MT-103-211, TOWER, and ALCANTARA); however, this trend was not observed in pediatric patients with R/R B-cell ALL. In conclusion, this analysis identified first salvage adults with Ph-negative R/R B-cell ALL as particularly well-suited for treatment with blinatumomab since survival outcomes from retrospective analyses reported in this patient subgroup were numerically better compared with those from pivotal studies without additional safety signals.

## Introduction

B-cell acute lymphoblastic leukemia (B-cell ALL) is caused by the malignant transformation and proliferation of lymphoid progenitor cells in the bone marrow, blood, and extramedullary sites.^1^ Despite improvements in treatment outcomes recorded in patients with newly diagnosed B-cell ALL, disease relapse remains the main cause of treatment failure. Survival rates following relapse range from <10.0% to about 25.0% in adults and from 30.0% to approximately 60.0% in pediatric patients, depending on the duration of the first remission, site of recurrence, and presence of certain recurrent cytogenetic or molecular abnormalities.^2–9^ Survival rates are worse in patients with second or later relapse. Historically, treatment options for patients with relapsed/refractory (R/R) B-cell ALL have been limited to conventional chemotherapy, followed by allogeneic hematopoietic stem cell transplantation (alloHSCT) in eligible patients. Due to the poor outcomes observed with chemotherapy, novel and more effective treatment options are needed.

Blinatumomab is a novel immunotherapy based on the BiTE® (bispecific T-cell engager) immuno-oncology platform that redirects CD3-positive T cells to serially engage and lyse CD19-expressing B cells, including leukemic blasts.^10,11^ Based on its tolerable safety profile and a high response rate, blinatumomab was first approved by the US Food and Drug Administration (FDA) for the treatment of R/R B-cell ALL in adults and pediatric patients and later for B-cell ALL with measurable residual disease (MRD; defined as the presence of ⩾10^−3^ leukemic blasts).^12^ Subsequently, blinatumomab was approved by the European Medicines Agency for the same indications.^13^ Since its approval, a large number of patients with R/R and MRD-positive B-cell ALL have been treated with blinatumomab. In addition to the results obtained from pivotal studies (defined as studies based on which the FDA approval was granted), data on survival in specific patient subgroups have been gathered in retrospective analyses, ethnicity/country-specific studies, and studies based on real-world evidence (RWE). Comparison of survival outcomes from these studies with those from pivotal studies may provide insight into patient subgroups that may benefit preferentially from blinatumomab. The objective of this review was to compare the survival outcomes – relapse-free survival (RFS) and overall survival (OS) – from the pivotal studies with those from retrospective analyses, single country- or ethnicity-specific studies, and RWE-based studies in patients with R/R B-cell ALL or patients with B-cell ALL with MRD treated with blinatumomab.

## Methods

**Figure 1.**
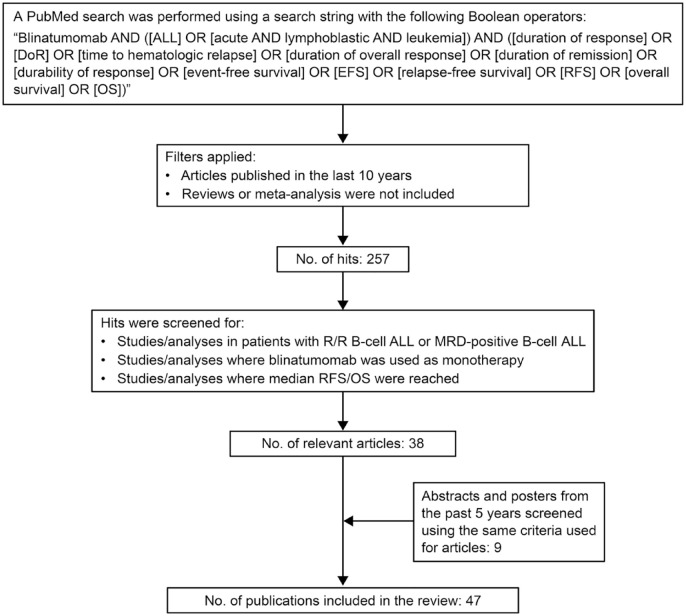
Flow chart for the strategy used for the literature search. ALL, acute lymphoblastic leukemia; DoR, duration of remission; EFS, event-free survival; OS, overall survival; R/R, relapsed/refractory; RFS, relapse-free survival.

A PubMed search was performed using a search string with the following Boolean operators: ‘Blinatumomab AND [(ALL) OR (acute AND lymphoblastic AND leukemia)] AND [(duration of response) OR (DoR) OR (time to hematologic relapse) OR (duration of overall response) OR (duration of remission) OR (durability of response) OR (event-free survival) OR (EFS) OR (relapse-free survival) OR (RFS) OR (overall survival) OR (OS)]’. The search identified 257 publications as of December 2022, of which 38 articles were found to report data for pivotal studies, retrospective analyses, ethnicity/country-specific studies, or RWE-based studies on median RFS or median OS in adult or pediatric patients with R/R B-cell ALL or MRD-positive B-cell ALL treated with blinatumomab (Figure 1). In addition, nine abstracts/posters presented at annual meetings of the American Society of Hematology (ASH), European Hematology Association (EHA), or American Society of Clinical Oncology (ASCO) based on studies sponsored by Amgen and those sponsored by institutions independent of Amgen were included. Articles published in the past 10 years (2013–2022) and abstracts published in the past 5 years (2018–2022) were considered. Reviews or meta-analyses were not included. Relevant articles and abstracts were grouped based on the type of patients assessed. Patients were subsequently grouped based on age, R/R disease with/without the presence of the Philadelphia chromosome (Ph), and the presence of MRD following frontline chemotherapy.

## Adults with R/R Ph-negative B-cell ALL

### Pivotal studies

MT-103-211 was a multicenter, single-arm, open-label phase II study that enrolled 189 patients [median age, 39 years (range, 18–79)] with R/R Ph-negative B-cell ALL^14^ who received blinatumomab by continuous intravenous (cIV) infusion at a target dose of 9 µg/day in the first week, followed by 28 µg/day for the remaining 3 weeks, repeated in 6-week cycles for up to five cycles. This study included patients with early relapse (defined as those in whom the relapse occurred within 12 months of the first remission or 12 months after receiving alloHSCT) or patients who did not respond to or relapsed after salvage therapy. After the first two cycles, 43% of the patients achieved complete remission (CR)/complete remission with partial hematological response (CRh). In patients who achieved CR/CRh, the median RFS was 5.9 months [95% confidence interval (CI), 4.8–8.3] and the median OS in patients treated with blinatumomab was 6.1 months (95% CI, 4.2–7.5).

Based on the encouraging results from this phase II study, the landmark randomized phase III study – TOWER (MT-103-311) – compared the efficacy and safety of blinatumomab with the investigator’s choice of standard-of-care chemotherapy in patients with R/R Ph-negative B-cell ALL.^15^ The study enrolled 405 patients who were randomized 2:1 to receive blinatumomab (*n* = 271) or salvage chemotherapy (*n* = 124). The dosage schedule was similar to the schedule used in the phase II MT-103-211 study^14^ and has been described in the primary publication.^15^ The percentage of patients who achieved CR/CRh/complete remission with incomplete hematological response (CRi) within 12 weeks of treatment initiation was significantly higher in the blinatumomab group [43.9% (95% CI, 37.9–50.0)] than in the chemotherapy group [24.6% (95% CI, 17.6–32.8), *p* < 0.001]. Among the patients who achieved CR/CRh/CRi, the median duration of remission (DoR) was 7.3 months (95% CI, 5.8–9.9) with blinatumomab compared with 4.6 months (95% CI, 1.8–19.0) with chemotherapy. The median OS was significantly longer with blinatumomab [7.7 months (95% CI, 5.6–9.6)] compared with chemotherapy [4.0 months (95% CI, 2.9–5.3), *p* = 0.01]. Frequently reported grade ⩾ 3 adverse events (AEs) with blinatumomab were neutropenia (37.8%) and infections (34.1%); the incidence of grade ⩾ 3 neurologic AEs was 9.4% and grade ⩾ 3 cytokine release syndrome (CRS) was 4.9%. Long-term follow-up of 56 patients from the pivotal TOWER study over a median duration of 60.0 months showed that the median OS for patients treated with blinatumomab was 7.6 months (Amgen data on file), which was similar to the median OS reported in the primary study.^15^

### Key retrospective analyses based on prospective clinical studies

Retrospective analyses of results from clinical studies have provided further insight into the RFS and OS in specific patient subgroups. A retrospective analysis by Stein *et al.*^16^ assessed data from 64 adult patients with R/R Ph-negative B-cell ALL with prior relapse following alloHSCT before enrollment in the above-mentioned phase II study (MT-103-211). Among patients receiving blinatumomab, 45.0% achieved CR/CRh within the first two treatment cycles; in these patients, the median RFS was 7.4 months (95% CI, 5.0–10.1) after a median follow-up of 12.4 months (Table 1 and Supplemental Figure 1). The median OS was 8.5 months (95% CI, 4.2–11.2) after a median follow-up of 16.6 months (95% CI, 12.4–23.3). The median RFS was 11.4 months (95% CI, 2.3–24.9) in patients who achieved CR/CRh and one previous relapse and 6.2 months [95% CI, 3.8–not estimable (NE)] in patients who had two or more previous relapses. Similar to the trend seen with RFS, the median OS was approximately twice as long for patients with one previous relapse compared with patients with two or more previous relapses [14.3 months (95% CI, 4.0–23.1) *versus* 6.5 months (95% CI, 3.5–9.3)].

**Table 1.** Median RFS and median OS in adult patients with R/R Ph-negative B-cell ALL treated with blinatumomab from pivotal studies, retrospective analyses, and single country or ethnicity-specific studies.

Reference	Study name/brief description	No. patients treated with blinatumomab	No. patients with CR/CRh^a^ (%)	Median RFS^b^, months (95% CI)	Median OS, months (95% CI)	Key safety results
Pivotal studies						
Topp *et al.*^14^	MT-103-211, phase II, single-arm study	189	81 (43.0)	5.9 (4.8–8.3)	6.1 (4.2–7.5)	The most common grade ⩾ 3 AEs were febrile neutropenia (25%), neutropenia (16%), and anemia (14%); 2% of patients had grade ⩾ 3 CRS; 12.7% of patients had grade ⩾ 3 neurologic AEs
Kantarjian *et al.*^15^	MT-103-311 (TOWER), phase III, randomized study	405 patients, 271 patients in the blinatumomab arm	119^c^ (43.9)	Not reported^d^	Blinatumomab arm, 7.7 (5.6–9.6) without censoring at HSCT and 6.9 (5.3–8.8) upon censoring at HSCT	Frequently reported grade ⩾ 3 AEs in the blinatumomab arm were neutropenia (37.8%) and infection (34.1%); incidence of grade ⩾ 3 neurologic AEs was 9.4% and of grade ⩾ 3 CRS was 4.9%
Clinical studies supportive of pivotal studies						
Topp *et al.*^17^	MT-103-206, phase II, single-arm study	36	25 (69.0)	No censoring of alloHSCT, 7.6 (4.5–9.5); censoring for alloHSCT, 7.9 (2.8–NE)	No censoring of alloHSCT, 9.8 (8.5–14.9); censoring for alloHSCT, 14.9 (8.2–21.9)	The most common grade ⩾ 3 AEs were transient leukopenia (13.9%) and thrombocytopenia (11.1%); 13.9% of patients had grade ⩾ 3 neurologic AEs and 5.6% of patients had grade ⩾ 3 CRS
Studies based on long-term follow-up						
Aboudalle *et al.*^18^	Long-term follow-up analysis of MT-103-211 at a single institution	35	19^c^ (54.0)	Not reported	10.6 (range, 0.2–68.0)	CRS (all grade 2) was reported in 17 (49.0%) patients; neurotoxicity (grades 1 and 2) was reported in 9 (26.0%) patients
Amgen data on file	Long-term follow-up of TOWER	75	Not reported	Not reported	7.6 (5.6–9.4)	Not reported
Topp *et al.*^19^	Long-term outcomes for MT-103-203 and MT-103-211	34 from MT-103-211	34^e^	Not reported for the overall patient population; 16.4 months for patients aged ⩽35 years and 15.9 months for patients aged >35 years	20.2 (7.1–NE) after blinatumomab and alloHSCT; NR for patients aged ⩽35 years and 15.9 months for patients aged >35 years	Not reported
Topp *et al.*^20^	Long-term outcomes of patients pooled from MT-103-206 and MT-103-211	259	123 (47.5)	7.7 (6.2–10.0)	7.5 (5.5–8.5); 18.1 (10.3–30.0) for patients in CR/CRh followed by alloHSCT	Not reported
Zugmaier *et al.*^21^	Long-term follow-up for MT-103-206	10	9 (90.0)	8.8 (5.7–13.2)	13.0 (8.5–21.9)	One patient had grade ⩾ 3 neurologic AE and one patient had grade ⩾ 3 CRS
Retrospective analyses based on clinical studies						
Stein *et al.*^16^	Exploratory analysis based on MT-103-211	64	29 (45.0)	7.4 (5.0–10.1) overall; 11.4 (2.3–24.9) for patients with one previous relapse; 6.2 (3.8–NE) for patients with two previous relapses	8.5 (4.2–11.2) overall; 14.3 (4.0–23.1) for patients with one previous relapse; 6.5 (3.5–9.3) for patients with two prior relapses	The most frequent grade ⩾ 3 AEs in patients with previous alloHSCT (*n* = 64) *versus* those without previous alloHSCT (*n* = 125) were febrile neutropenia (20.3% *versus* 28.8%), neutropenia (21.9% *versus* 12.8%), and anemia (17.2% *versus* 12.8%). Grade ⩾ 3 neurologic AEs in patients with previous alloHSCT *versus* those without previous alloHSCT were 15.6% *versus* 12.0%. Grade ⩾ 3 CRS in patients with previous alloHSCT *versus* those without previous alloHSCT were 3.1% *versus* 0.8%
Dombret *et al.*^22^	Subgroup analysis of TOWER (first salvage *versus* second or later salvage)	104 patients treated with blinatumomab as first salvage and 167 patients treated with blinatumomab as second or later salvage	Blinatumomab as first salvage, 53^c^ (51.0); blinatumomab as second or later salvage, 66^c^ (39.5)	First salvage, 1.9 (0.0–6.5); second or later salvage, 0.0 (NE–NE)	First salvage, 11.1 (8.2–NE), 9.6 (7.0–15.6) if censored for alloHSCT; second or later salvage, 5.1 (3.2–7.1), 4.7 (3.2–7.1) if censored for alloHSCT	First salvage Grade ⩾ 3 AEs of interest were cytopenia (68.0%), neutropenia (44.7%), and infections (25.2%). Grade ⩾ 3 neurologic event (8.7%) and grade ⩾ 3 CRS (3.9%) Second or later salvage Grade ⩾ 3 AEs of interest were cytopenia (67.7%), neutropenia (46.3%), and infections (32.9%). Grade ⩾ 3 neurologic events (9.8%) and grade ⩾ 3 CRS (5.5%)
Topp *et al.*^23^	Pooled analysis from Study MT-103-206, Study MT-103-211, and TOWER	165 patients treated with blinatumomab as first salvage; 367 patients treated with blinatumomab as second or later salvage	Blinatumomab as first salvage, 89^c^ (54.0); blinatumomab as second or later salvage, 150^c^ (41.0)	Blinatumomab as first salvage, 10.1 (7.4–18); blinatumomab as second or later salvage, 7.3 (5.7–9.6)	Blinatumomab as first salvage, 10.4 (8.3–14.3); blinatumomab as second salvage, 5.7 (4.3–7.1)	First salvage Grade ⩾ 3 anemia (20.0%); grade ⩾ 3 neutropenia (20.0%); grade ⩾ 3 febrile neutropenia (18.0%); grade ⩾ 3 neurologic AEs (13.0%); grade ⩾ 3 CRS (28.0%) Second or later salvage Grade ⩾ 3 anemia (15.0%); grade ⩾ 3 neutropenia (15.0%); grade ⩾ 3 febrile neutropenia (24.0%); grade ⩾ 3 neurologic AEs (15.0%); grade ⩾ 3 CRS, (38.0%)
Rambaldi *et al.*^24^	Subgroup analysis of TOWER	86 patients received blinatumomab consolidation; 36 patients received blinatumomab consolidation + maintenance	71^c^ (82.6) for patients who received blinatumomab consolidation; 31^c^ (86.1) for patients who received blinatumomab maintenance	Consolidation group, 7.6 months (95% CI, 3.7–11.6) *versus* no-consolidation group, 8.8 months (95% CI, 0.0–10.4) Maintenance group, 14.5 months (95% CI, 7.1–21.9) *versus* no-maintenance group (9.8 months, 95% CI, 8.5–11.1)	Consolidation group, 16.6 months (95% CI, 13.6 to 19.6) *versus* no-consolidation group, 13.0 months (95% CI, NE) Maintenance group, NR *versus* no-maintenance group 15.5 months (95% CI, NE)	Grade ⩾ 3 neurologic AEs were reported in 11.1% of patients in induction, 0% in consolidation, and 11.1% in maintenance, whereas grade ⩾ 3 CRS was reported in 5.6% of patients in induction, 0% in consolidation, and 2.8% in maintenance
Jabbour *et al.*^25^	Subgroup analysis of TOWER	65 patients received blinatumomab + alloHSCT; 206 patients received blinatumomab but no alloHSCT	50^c^ (77.0)	Patients with CR/CRh/CRi and no alloHSCT, 6.7 (5.28–8.09); patients with CR/CRh/CRi and alloHSCT 8.8 (5.46–12.05)	Blinatumomab but no alloHSCT, 10.1 (8.5–11.8); blinatumomab + alloHSCT, NE (NE–NE)	Not reported
Gökbuget *et al.*^26^	Subgroup analysis of MT-103-211	Overall MRD-evaluable, 90; MRD responders, 75; MRD non-responders, 15	NA	MRD responders, 9.0 (6.2–14.6); MRD non-responders, 2.3 (1.2–7.5)	MRD responders, 20.6 (10.9–30.5); MRD non-responders, 12.5 (2.0–23.9)	The most frequent grade ⩾ 3 AEs in the MRD responders *versus* MRD non-responders were neutropenia (21.0% *versus* 20.0%) and febrile neutropenia (19.0% *versus* 20.0%)
Kantarjian *et al.*^27^	Subgroup analysis of MT-103-206 and MT-103-211	36 adults aged ⩾65 years; 225 adults aged <65 years	20 (56.0) for those aged ⩾65 years; 104 (46.0) for those aged <65 years	Adults ⩾65 years, 7.4 (2.7–23.1); adults <65 years, 7.4 (6.1–9.0)	Adults ⩾65 years, 5.5 (4.2–13.5); adults <65 years, 7.6 (5.8–8.6)	The most common grade ⩾ 3 AEs in patients aged ⩾65 *versus* <65 years were febrile neutropenia (22.0% *versus* 23.0%), leukopenia (14.0% *versus* 7.0%), and anemia (8.0% *versus* 13.0%). In patients aged ⩾65 *versus* <65 years, the incidence of grade ⩾ 3 neurologic AEs was 28.0% *versus* 13.0% and grade ⩾ 3 CRS was 3.0% *versus* 2.0%
Single-center clinical studies						
Aldoss *et al.*^28^	Single-center analysis (City of Hope Medical Center)	65	33 (51.0)	6.3	10.7 (not reported) for patients with CR/CRh	Grade ⩾ 2 CRS, 33.0% in patients with CR/CRh and 9.0% in patients without CR/CRh
Yoon *et al.*^29^	Single-center analysis (The Catholic University of Korea)	32	22 (68.8)	Not reported	18.2 (not reported)	Grade ⩾ 3 neutropenia, 59.3%; grade ⩾ 3 thrombocytopenia, 56.2%; febrile neutropenia, 53.1%; no grade ⩾ 3 CRS or grade ⩾ 3 neurotoxicity
Country/ethnicity-specific studies						
Zhou *et al.*^30,31^	Chinese patients	90	41 (46.0)	4.3 (3.2–9.4)	9.2 (6.5–11.7)	Neutropenia and febrile neutropenia (58.9%), infection (42.2%), elevated liver enzymes (22.2%)
Jung *et al.*^32^	Korean patients	50	22 of 49 evaluable patients (44.9)	3.3 (range, 2.1–4.6)	7.5 (range, 5.3–9.7)	The most common grade ⩾ 3 AEs were febrile neutropenia (34.0%), neutropenia (66.0%), and thrombocytopenia (62.0%). The incidence of grade ⩾ 3 CRS was 4.0%
Kiyoi *et al.*^33^	Study 20130265 (Study 265), Japanese patients	Five patients in phase Ib and 21 patients in phase II	8 (38.0) in the phase II part	5.0 (3.5–6.4)	NE (7.4–NE)	In the phase II part, the most common grade ⩾ 3 AEs were cytopenia (81.0%), neutropenia (76.0%), and infection (38.0%). The incidence of grade ⩾ 3 neurologic AEs was 5.0% and grade ⩾ 3 CRS was 5.0%
Kobayashi *et al.*^34^	Pooled analysis of Asian patients from TOWER and Study 265	45	20 (44.4)	8.9 (3.8–10.7)	11.9 (9.9–17.1)	The most frequent grade ⩾ 3 AEs of interest were cytopenia (63.6%), neutropenia (59.1%), and infections (43.2%). The incidence of grade ⩾ 3 neurologic AEs was 9.1% and grade ⩾ 3 CRS was 2.3%

a Achieved within the first 2 cycles of treatment with blinatumomab.

b Median RFS was calculated in patients with CR/CRh or CR/CRh/CRi.

c Patients with CR/CRh/CRi.

d 6-month RFS estimates were 31% *versus* 12% in the blinatumomab *versus* chemotherapy groups; hazard ratio for an event of relapse after achieving CR, CRh, CRi, or death, 0.55; 95% CI, 0.43–0.71; *p* < 0.001.

e Continuous hematologic CR. CR is defined as ⩽5% bone marrow and no evidence of disease, platelet count >100,000 per µL, and absolute neutrophil count >1000 per µL. CRh is defined as ⩽5% bone marrow blasts and no evidence of disease, platelet count >50,000 per µL, and absolute neutrophil count >500 per µL. CRi is defined as ⩽5% bone marrow blasts and no evidence of disease, platelet count of >100,000 per µL or absolute neutrophil count of >1000 per µL.

AE, adverse event; ALL, acute lymphoblastic leukemia; alloHSCT, allogeneic hematopoietic stem cell transplantation; CI, confidence interval; CR, complete remission with full hematologic recovery; CRh, complete remission with partial hematologic recovery; CRi, complete remission with incomplete hematologic recovery; CRS, cytokine release syndrome; HSCT, hematologic stem cell transplantation; MRD, measurable residual disease; NA, not applicable; NE, not estimable; NR, not reached; OS, overall survival; Ph, Philadelphia chromosome; RFS, relapse-free survival; R/R, relapsed / refractory.

In two other retrospective analyses, patients with R/R B-cell ALL treated with blinatumomab were assessed for survival outcomes based on the status of first *versus* later relapse. In the first report by Dombret *et al.*, which analyzed data from 104 and 167 adult patients treated with blinatumomab as first or second/later salvage therapy, respectively, from the phase III TOWER study, the median OS was numerically longer in patients treated in first salvage [11.1 months (95% CI, 8.2–not reached (NR))] compared with those receiving blinatumomab as second/later salvage therapy [5.1 months (95% CI, 3.2–7.1)].^22^ A similar trend was noted with the DoR in patients who achieved CR/CRh/CRi in response to blinatumomab. The median DoR was 10.7 months (95% CI, 5.6–NE) compared with 6.2 months (95% CI, 3.8–9.6) in patients treated with blinatumomab as first or second/later salvage therapy, respectively. The incidence of grade 3 AEs of interest reported in at least 3% of patients (61.2% *versus* 67.7%), grade 3 neurologic AEs (7.8% *versus* 9.1%), and grade 3 CRS (3.9% *versus* 4.9%) were comparable in patients who received blinatumomab as first *versus* second/later salvage.

In the second retrospective analysis by Topp *et al.*,^23^ data from two phase II studies and the phase III TOWER study were analyzed. Patients who received blinatumomab as first salvage compared with second/later salvage therapy demonstrated higher rates of CR/CRh after two cycles [54.0% *versus* 41.0%; odds ratio (OR), 0.59; *p* = 0.005] and had a longer median OS [10.4 *versus* 5.7 months; hazard ratio (HR), 1.58; *p* < 0.001]. The median RFS in patients who received blinatumomab as first salvage compared with second/later salvage therapy was numerically longer (10.1 *versus* 7.3 months; HR, 1.38; *p* = 0.061), the difference was not statistically significant. The safety profile of blinatumomab was generally similar between the two treatment groups (grade ⩾ 3 neurologic AEs, 13.0% *versus* 15.0%; grade ⩾ 3 CRS, 28.0% *versus* 38.0%; the latter event slightly greater in the blinatumomab as second/later salvage therapy group). These results indicate that although treatment with blinatumomab was beneficial when administered as either first or second/later salvage therapy, the effect of treatment was more favorable when blinatumomab was administered as first salvage therapy.

AlloHSCT following the achievement of CR has been suggested as a potentially curative approach for patients with R/R B-cell ALL. Jabbour *et al.* performed a retrospective analysis^25^ to assess the effect of alloHSCT on survival in patients treated with blinatumomab in the phase III TOWER study. Of the 97 patients who underwent on-study alloHSCT, 65 received prior treatment with blinatumomab, of whom 77.0% had CR/CRh/CRi. With a median follow-up of 7.2 months, the median RFS in patients who achieved CR/CRh/CRi in response to treatment with blinatumomab followed by subsequent on-study alloHSCT was 8.8 months (95% CI, 5.5–12.1) compared with a median RFS of 6.7 months (95% CI, 5.3–8.1) in patients treated with blinatumomab without subsequent on-study alloHSCT (*p* = 0.97). The median OS was not reached in patients with CR/CRh/CRi who received subsequent alloHSCT compared with 16.0 months for those with CR/CRh/CRi but no alloHSCT (OR, 1.17; 95% CI, 0.5–2.5; *p* = 0.69). There was insufficient evidence to detect a difference in the survival outcomes between patients who did or did not receive on-study alloHSCT following treatment with blinatumomab. Survival was found to be dependent on response to treatment with blinatumomab regardless of whether patients had on-study alloHSCT.

A retrospective analysis of patients from the phase II study MT-103-211^17^ was performed by Gökbuget *et al.*^26^ to assess the effect of MRD response (<10^−4^ detectable leukemic blasts) on OS in a subset of 90 patients treated with blinatumomab who were MRD-evaluable (achieved CR/CRh during the first two cycles and had evaluable MRD data). MRD response was achieved in 75 of the 90 patients (83.3%). The median RFS for MRD responders [9.0 months (95% CI, 6.2–14.6)] was significantly longer compared with the median RFS for MRD non-responders [2.3 months (95% CI, 1.2–7.5); *p* = 0.013 (log-rank test) and *p* = 0.004 (Wilcoxon)]. In addition, the median OS was significantly longer for MRD responders [20.6 months (95% CI, 10.9–30.5)] compared with that for MRD non-responders [12.5 months (95% CI, 2.0–23.9); *p* = 0.03 (log-rank test) and *p* = 0.05 (Wilcoxon)]. No significant difference in the incidence of AEs was observed in MRD responders *versus* MRD non-responders.

### Key country-specific or ethnicity-specific studies

Prospective studies aimed at demonstrating the efficacy of blinatumomab in patients with R/R B-cell ALL from a specific country or a specific ethnic background have been conducted. A multicenter, single-arm study that enrolled 90 heavily pretreated patients with R/R Ph-negative B-cell ALL from China showed that at a dosing similar to that followed in the pivotal studies (MT-103-211 and TOWER), the rate of CR/CRh within two cycles of blinatumomab was 45.6% (95% CI, 35.0–56.4).^30,31^ The median RFS in patients with CR/CRh was 4.3 months (95% CI, 3.2–9.4), and the median OS in all patients treated with blinatumomab was 9.2 months (95% CI, 6.5–11.7). A phase Ib/II study conducted in Japan in patients with R/R Ph-negative B-cell ALL showed that 8 of 21 patients (38.0%) in the phase II part of the study achieved CR/CRh within two cycles of blinatumomab treatment. The median RFS among these patients was 5.0 months (95% CI, 3.5–6.4) and the median OS was NE (95% CI, 7.4–NE).^33^ Another study that evaluated the safety and efficacy of blinatumomab in Korean patients with R/R Ph-negative B-cell ALL showed that 22 of the 49 evaluable patients (44.9%) achieved CR/CRh.^32^ The median RFS in patients with CR/CRh was 7.5 months, which was significantly longer than the median RFS of 2.0 months in patients who did not achieve CR/CRh (*p* < 0.001). The median OS was significantly longer in patients with CR/CRh compared with those without CR/CRh (8.1 *versus* 5.2 months, *p* < 0.001). A similar trend was seen in patients who underwent alloHSCT after treatment with blinatumomab; these patients had a median RFS of 7.5 *versus* 2.4 months in patients with no alloHSCT (*p* < 0.001) and the median OS was 7.5 *versus* 5.2 months (*p* = 0.058). A retrospective analysis of 45 Asian patients with R/R Ph-negative B-cell ALL pooled from two studies – TOWER^15^ and the phase Ib/II study in Japanese patients reported by Kiyoi *et al.*^33^ – showed that the median RFS in patients who achieved CR/CRh in response to blinatumomab within the first 12 weeks was 8.9 months (95% CI, 3.8–10.7) and the median OS was 11.9 months (95% CI, 9.9–17.1).^34^ Overall, the median RFS and median OS in Asian patients with R/R Ph-negative B-cell ALL were comparable to the median RFS and median OS reported in pivotal clinical studies (MT-103-211 and TOWER).

## Adults with R/R Ph-positive B-cell ALL

### Pivotal study

The presence of the Ph is associated with poor outcomes in patients with R/R B-cell ALL.^35^ The open-label, multicenter, single-arm, phase II study – ALCANTARA – enrolled 45 adult patients with R/R Ph-positive B-cell ALL who had either progressed or not responded to second- or later-generation tyrosine kinase inhibitors (TKIs) or those who were intolerant to TKI therapy (Table 2 and Supplemental Figure 2).^36^ A total of 16 patients (35.6%) achieved CR/CRh within the first two cycles; of these, 88.0% had a complete MRD-negative response (defined as no detectable polymerase chain reaction amplification of *BCR-ABL1* genes at a sensitivity ⩾10^−5^). With a median follow-up of 9.0 months, the median RFS was 6.7 months (95% CI, 4.4–NE) and the median OS was 7.1 months (95% CI, 5.6–NE). None of the patients experienced grade ⩾ 3 CRS and the incidence of grade ⩾ 3 neurologic AEs was 6.7%. The long-term follow-up study of the 45 patients enrolled in the primary study showed that the median RFS was 6.8 months (95% CI, 4.4–NE) at a median follow-up of 16.1 months and the median OS was 9.0 months (95% CI, 5.7–13.5) at a median follow-up of 25.1 months.^37^ The median OS in patients with CR [19.8 months (95% CI, 12.1–NE)] was longer than in those without CR [6.0 months (95% CI, 2.9–7.1)].

**Table 2.** Median RFS and median OS in adult patients with R/R Ph-positive B-cell ALL treated with blinatumomab from pivotal studies.

Reference	Study name/descriptor	No. patients treated with blinatumomab	No. patients with CR/CRh (%)	Median RFS^a^, months (95% CI)	Median OS, months (95% CI)	Key safety results
Pivotal study						
Martinelli *et al.*^36^	ALCANTARA	45	16 (36.0)	6.7 (4.4–NE); 6.8 (4.4–NE) in patients who achieved CR/CRh + complete MRD response^b^; 5.5 (3.6–NE) for patients younger than age 55 years; 6.7 (3.8–NE) for patients aged 55 years or older	7.1 (5.6–NE) with or without censoring for alloHSCT	The most common grade ⩾ 3 AEs were febrile neutropenia (26.7%), thrombocytopenia (26.7%), and anemia (17.8%). The incidence of grade ⩾ 3 CRS was 0% and grade ⩾ 3 neurologic AEs was 6.7%
Martinelli *et al.*^37^	Final analysis of ALCANTARA	45	16 (35.6)	6.8 (4.4–NE)	9.0 (5.7–13.5) overall; 23.0 months (12.6–NE) in patients with CR/CRh	Grade ⩾ 3 AEs included febrile neutropenia (11.0%); elevated alanine aminotransferase (11.0%); no grade ⩾ 3 CRS (0%); neurologic grade ⩾ 3 AEs (13.0%)

a Median RFS was calculated in patients with CR/CRh. CR is defined as ⩽5% bone marrow and no evidence of disease, platelet count >100,000 per µL, and absolute neutrophil count >1000 per µL. CRh is defined as ⩽5% bone marrow blasts and no evidence of disease, platelet count of >50,000 per µL, and absolute neutrophil count of >500 per µL.

b Complete MRD response was defined as no detectable PCR amplification of *BCR-ABL1* genes (sensitivity ⩾10^−5^) as assessed by a central laboratory.

AE, adverse event; ALL, acute lymphoblastic leukemia; alloHSCT, allogeneic hematopoietic stem cell transplantation; CI, confidence interval; CR, complete remission with full hematologic recovery; CRh, complete remission with partial hematologic recovery; CRS, cytokine release syndrome; MRD, measurable residual disease; NE, not estimable; OS, overall survival; Ph, Philadelphia chromosome; RFS, relapse-free survival; R/R, relapsed / refractory.

### Retrospective analyses or country-specific/ethnicity-specific studies

No retrospective analyses aimed at the assessment of RFS and OS in specific patient subgroups from within the pivotal ALCANTARA study or other country/ethnicity-specific studies in patients with R/R Ph-positive B-cell ALL have been reported.

## Adult patients with MRD-positive B-cell ALL

### Pivotal study

A phase II study – BLAST (MT-103-203) – evaluated the efficacy and tolerability of blinatumomab in patients with MRD-positive B-cell ALL with first or later CR at baseline.^38^ MRD positivity was defined as the presence of ⩾10^−3^ leukemic blasts. Patients found to be MRD positive after receiving a minimum of three blocks of intensive chemotherapy were eligible to receive treatment with blinatumomab. Blinatumomab was administered as cIV infusion at a dose of 15 µg/m^2^/day over 4 weeks, followed by a treatment-free period of 2 weeks, which was defined as one 6-week treatment cycle. Patients with Ph-positive B-cell ALL were excluded from the survival analysis. Of the 113 evaluable patients, 88 (77.9%) had a complete MRD response (defined as no detectable leukemic blasts) at the end of cycle 1 of blinatumomab; two additional patients showed an MRD response following cycle 2. The median RFS in all patients was 18.9 months (95% CI, 12.3–35.2) and the median OS was 36.5 months (19.8–NR; Table 3 and Supplemental Figure 3). In patients with complete MRD response, the median RFS was 23.6 months (95% CI, 17.4–NR) and the median OS was 38.9 months (95% CI, 33.7–NR). The most frequent grade ⩾ 3 AE in patients treated with blinatumomab was neutropenia (26.0%). The incidence of grade ⩾ 3 neurologic AEs was 21.7% and that of grade ⩾ 3 CRS was 1.7%. Results from the extended follow-up over a median duration of 59.8 months showed that the median OS for patients with a complete MRD response was significantly longer than for those without a complete MRD response [NR (95% CI, 29.5–NR) *versus* 14.4 months (95% CI, 3.8–32.3); log-rank *p* = 0.002].^39^ Furthermore, in patients who were treated with blinatumomab after the first remission, the median OS was significantly longer [NR (95% CI, 29.5–NR)] among patients with a complete MRD response in cycle 1 compared with patients without a complete MRD response [10.6 months (95% CI, 2.7–39.7); *p* = 0.008 (log-rank)]. Among patients in second or later remission treated with blinatumomab, the median OS was 38.8 months [95% CI, 13.9–NR] in those with a complete MRD response *versus* 16.0 months [95% CI, 2.0–NR] in those without a complete MRD response [*p* = 0.14 (log-rank)]. The rate of non-relapse mortality in patients who received alloHSCT after treatment with blinatumomab was 36.5%. This was due to the high median age of the patients and the high rate of mismatched donors used for the allograft. Most patients who did not receive a transplant relapsed (72.2%) and nearly half of them received a transplant after subsequent relapse. Regardless of the alloHSCT status, the 5-year survival rate for patients who had a complete MRD response was 50.0%. No parameters that allowed prediction of this long-term response were identified.

**Table 3.** Median RFS and median OS in adult patients with MRD-positive B-cell ALL treated with blinatumomab from pivotal studies.

Reference	Study name/descriptor	No. patients treated with blinatumomab	No. MRD responders^a^ (%)	Median RFS^b^, months (95% CI)	Median OS, months (95% CI)	Key safety results
Pivotal study						
Gökbuget *et al.*^38^	MT-103-203 (BLAST)	116	113 88 (77.9) had a complete MRD response^c^	18.9 (12.3–35.2) Complete MRD responders^c^, 23.6 (17.4–NR); MRD non-responders, 5.7 (1.6–13.6); patients in first CR + complete MRD response^c^, NR; patients in ⩾2 CR + complete MRD response^c^, 13.9	36.5 (19.2–NE) Complete MRD responders^c^, 38.9 (33.7–NR); MRD non-responders, 12.5 (3.2–NR); patients in first remission after blinatumomab, 36.5 (20.6–NR); patients in second or later remission after blinatumomab, 19.1 (11.9–NR)	Grade ⩾ 3 neutropenia, 15.5%; grade ⩾ 3 pyrexia, 7.8%; grade ⩾ 3 leukopenia, 6.0%; grade ⩾ 3 neurologic AEs, 13.0%; grade ⩾ 3 CRS, 1.7%
Long-term follow-up studies						
Gökbuget *et al.*^39^	MT-103-203 (BLAST)	116	107 84 (73.3) had a complete MRD response	Not reported	36.5 (22.0–NR); patients in first remission after blinatumomab, 41.2 (23.5–NR); patients in second or later remission after blinatumomab, 23.1 (15.4–NR) NR (29.5–NR); blinatumomab use in first remission, NR (29.5–NR); blinatumomab use in second or later remission, 38.8 (13.9–NR); CR + alloHSCT, NR; CR + no alloHSCT, 56.4 months (15.6–NR)	Not reported
Topp *et al.*^19^	Long-term outcomes for MT-103-203 and MT-103-211	74 from MT-103-203 with CR + alloHSCT	Not reported	Not reported	Overall, 36.7 (18.0–NE); MRD complete responders, NR (25.7–NR); MRD non-responders, 16.1 months (1.1–NR); patients ⩽35 years, NR; patients >35 years, 25.7	Not reported

a MRD response was defined as either a complete MRD response or the presence of ⩽10^−4^ detectable leukemic blasts.

b Median RFS was calculated in patients with CR/CRh/CRi and an MRD response at cycle 1 of blinatumomab. CR is defined as ⩽5% bone marrow and no evidence of disease, platelet count >100,000 per µL, and absolute neutrophil count >1000 per µL. CRh is defined as ⩽5% bone marrow blasts and no evidence of disease, platelet count >50,000 per µL, and absolute neutrophil count >500 per µL. CRi is defined as ⩽5% bone marrow blasts and no evidence of disease, platelet count of >100,000 per µL or absolute neutrophil count of >1000 per µL.

c Complete MRD response was defined as no target amplification with a minimum sensitivity of 10^−4^.

AE, adverse event; ALL, acute lymphoblastic leukemia; alloHSCT, allogeneic hematopoietic stem cell transplantation; CI, confidence interval; CR, complete remission with full hematologic recovery; CRh, complete remission with partial hematologic recovery; CRi, complete remission with incomplete hematologic recovery; CRS, cytokine release syndrome; MRD, measurable residual disease; NE, not estimated; NR, not reached; OS, overall survival; RFS, relapse-free survival.

### Retrospective analyses or country-specific/ethnicity-specific studies

No retrospective analyses aimed at the assessment of RFS and OS in specific patient subgroups from the pivotal BLAST study or other country-specific/ethnicity-specific studies in patients with MRD-positive B-cell ALL have been reported.

## Key studies based on RWE in adult patients

### Patients with R/R B-cell ALL

Reports based on an analysis of RWE have provided further support to the efficacy of blinatumomab observed in clinical studies. Badar *et al.* retrospectively analyzed RWE in a large cohort of 239 patients with B-cell ALL from 11 academic institutions in the US (Table 4).^40^ Overall, 227 patients received blinatumomab for R/R B-cell ALL, of which 55 were Ph-positive and included patients who received blinatumomab + TKI. The percentage of patients with R/R B-cell ALL who achieved CR/CRi was 67.0% after treatment with blinatumomab. The median RFS in patients with R/R B-cell ALL (Ph negative, *n* = 172; Ph positive, *n* = 55) was 32.1 months (95% CI, 9.5–NR), which was numerically about five times longer than the median RFS reported in the pivotal MT-103-211 study by Topp *et al.*^14^ (5.9 months; median RFS was not reported for TOWER). In the subgroup of patients who were Ph positive and treated with blinatumomab alone (*n* = 32), the median RFS was 32.0 months, which was also numerically about four times longer than the median RFS reported in the final analysis for ALCANTARA (6.8 months). With a median follow-up of 14.0 months, the median OS in patients with R/R (Ph negative, *n* = 172; Ph positive, *n* = 55) B-cell ALL treated with blinatumomab was 12.7 months (95% CI, 9.2–17.9), which was about twice the median OS reported for patients with R/R Ph-negative B-cell ALL from the pivotal studies – phase II study by Topp *et al*. (6.1 months) and TOWER (7.7 months). Among patients with R/R Ph-positive B-cell ALL who received blinatumomab alone, the median OS was 13.1 months, which was numerically longer than the median OS reported among patients from the final analysis for ALCANTARA (9.0 months).

**Table 4.** Median RFS and median OS from real-world evidence studies in adults with R/R or MRD-positive B-cell ALL treated with blinatumomab.

Reference	No. patients treated with blinatumomab (Ph-chromosome negative/positive)	No. patients with CR/CRh/CRi or MRD negative (%)	Median RFS^a^, months (95% CI)	Median OS, months (95% CI)	Key safety results
R/R B-cell ALL					
Boissel *et al.*^41^	106 (all patients were Ph negative)	54^b^ (50.9)	11.0 (8.2–15.4)	12.2 (7.3–24.2); 9.5 (7.1–24.2) upon censoring for alloHSCT; 17.8 (range, 1.1–17.8) in patients who had CR/CRh/CRi + alloHSCT	Not reported
Apel *et al.*^42^	21 (two were Ph positive; not reported for Ph negative)	11^c^ (52.0)	8.7 (Not reported)	15.2 (Not reported)	Grade ⩾ 3 neurologic AEs, one patient; grade ⩾ 3 cytokine storm, two patients
Cabannes-Hamy *et al.*^43^	38 (11 were Ph positive; not reported for Ph negative)	26^c^ (68.0)	14.6 (5.7–41.6)	10.3 (7.1–40.7)	Grade ⩾ 3 neurologic AEs, one patient; grade ⩾ 3 CRS, one patient
Badar *et al.*^40^	227 166 were Ph negative 32 were Ph positive and treated with blinatumomab alone (no TKI)	149^d^ (67.0) Not reported 22^d^ (69.0)	32.1 (9.5 to NR) Not reported 32.0 (not reported)	12.7 (9.2–17.9), 28.5 in patients with CR/CRi Not reported 13.1 (not reported)	Grade ⩾ 3 CRS (3.0%); grade ⩾ 3 neurologic AEs (7.0%); grade ⩾ 3 hepatotoxicity (10.0%) Not reported Not reported
Badar *et al.*^44^	221 166 were Ph negative 54 were Ph positive	128^d^ (58.0) 89^d^ (61.0) 39^d^ (76.5)	Not reported Not reported Not reported	15.0 (10.3–23.7) 10.9 (8.1–17.9) NR	Not reported Not reported Not reported
Chiaretti *et al.*^45^	34 (all were Ph positive)	14^c^ (41.2)	6.7 (IQR, 3.3–18.2)	16.3 (IQR, 4.6–NE)	Not reported
MRD-positive B-cell ALL					
Boissel *et al.*^46^	109 (83 patients were Ph negative; 26 patients were Ph positive)	66^e^ (82.5)	27.6 (range, 0.4–33.0); 33.0 (range, 0.4–33.0) upon censoring for alloHSCT	NR (range, 1.8–34.8); 4.0 (range, 2.6–13.7) upon censoring for alloHSCT	Not reported
Badar *et al.*^40^	12 (six patients were Ph positive)	9^e^ (75.0)	NR	34.7 (8.8–34.7)	Not reported

a Median RFS was calculated in patients with CR/CRh/CRi. CR is defined as ⩽5% bone marrow and no evidence of disease, platelet count >100,000 per µL, and absolute neutrophil count >1000 per µL. ⩽5% bone marrow blasts and no evidence of disease, platelet count >50,000 per µL, and absolute neutrophil count >500 per µL. CRi is defined as ⩽5% bone marrow blasts and no evidence of disease, platelet count of >100,000 per µL or absolute neutrophil count of >1000 per µL.

b Patients with CR/CRh/CRi.

c Patients with CR.

d Patients with CR/CRi.

e Patients who were MRD negative (defined as <10^−4^ leukemic blasts).

AE, adverse event; ALL, acute lymphoblastic leukemia; alloHSCT, allogeneic hematopoietic stem cell transplantation; CI, confidence interval; CR, complete remission with full hematologic recovery; CRh, complete remission with partial hematologic recovery; CRi, complete remission with incomplete hematologic recovery; CRS, cytokine release syndrome; IQR, interquartile range; MRD, measurable residual disease; NE, not estimable; NR, not reached; OS, overall survival; Ph, Philadelphia chromosome; RFS, relapse-free survival; R/R, relapsed/refractory; TKI, tyrosine kinase inhibitor.

Results from another large observational study (NEUF), where patients were enrolled in the expanded access program in selected European countries – France, Italy, Russia, Spain, and the UK – were based on the analysis of RWE from 106 patients with R/R Ph-negative B-cell ALL. In patients who achieved CR/CRh/CRi, the median RFS was 11.0 months (range, 0.0–15.4).^41^ The median OS was 12.2 months (range, 0.2–24.6; 9.5 months upon censoring for alloHSCT) compared with 6.1 months in the phase II study by Topp *et al.* 7.7 months in TOWER. In patients who underwent alloHSCT following CR/CRh/CRi in response to blinatumomab, the median OS was 17.8 months (range, 1.1–17.8). The NEUF study also included an analysis of RWE from 34 patients with R/R Ph-positive B-cell ALL. The results showed that among the 14 patients who achieved a CR, the median RFS was 6.7 months [interquartile range (IQR), 3.3–18.2] upon a median follow-up of 21.7 months and the median OS was 16.3 months (IQR, 4.6–NE) upon a median follow-up of 13.0 months.^45^ Overall, the median RFS and median OS assessed in patients with R/R B-cell ALL from studies based on analysis of real-world data were numerically longer compared with those reported in the pivotal studies.

### Patients with MRD-positive B-cell ALL

In the retrospective analysis of RWE by Badar *et al.* discussed earlier, 12 patients received blinatumomab for MRD-positive B-cell ALL and 8 (75.0%) were MRD negative after treatment.^40^ The median RFS for patients who received blinatumomab for MRD-positive B-cell was NR (54.0% of patients were MRD negative at the end of 2-year follow-up) and the median OS was 34.7 months (95% CI, 8.8–34.7; median follow-up, 6.0 months), which was similar to the median OS reported for patients from the pivotal BLAST study (38.9 months). Another report based on an analysis of real-world data from 109 patients with MRD-positive B-cell ALL enrolled in the NEUF study showed a median RFS of 27.6 months, which was comparable with the median RFS reported in the pivotal BLAST study (23.6 months),^38^ while the median OS was NR.^46^ Thus, the median RFS and median OS in patients with MRD-positive B-cell ALL treated with blinatumomab reported in studies based on RWE were comparable with the median RFS and median OS reported in BLAST.

## Pediatric patients with R/R or MRD-positive B-cell ALL

Treatment of R/R B-cell ALL in pediatric patients has remained a challenge, with survival rates lower than those observed at initial diagnosis. An initial study by Handgretinger *et al.* in three pediatric patients with post-transplant relapsed B-cell ALL showed that these patients achieved a CR when treated with blinatumomab in addition to infusion with donor T cells.^47^ Another follow-up report showed that of the nine patients treated with blinatumomab for post-HSCT relapse, six achieved CR.^48^

### Pivotal study

An open-label, multicenter, single-arm, phase I/II study, MT-103-205, was conducted in pediatric patients with R/R B-cell ALL to assess the optimal dosage, safety, and efficacy of blinatumomab.^49^ Eligible patients were <18 years of age (2–17 years of age in the dose-escalation phase) with B-cell ALL with >25% bone marrow blasts who were in second or later relapse or relapse following alloHSCT or refractory to other treatments and had failed a full standard induction or reinduction regimen (if in first relapse).^49^ In all, 49 patients were enrolled in phase I of the study to determine the maximum tolerated dose. An additional 26 patients were enrolled to further assess the pharmacokinetic profile of blinatumomab at the selected dose. Overall, 70 patients from the phase I (*n* = 26) and phase II (*n* = 44) parts of the study were treated with a stepwise dosage of 5/15 μg/m^2^/day; 27 patients (39.0%) achieved CR within the first two cycles of blinatumomab treatment, 52.0% of whom were MRD negative. With a median follow-up of 23.1 months, the median RFS for patients who achieved CR was 4.4 months (95% CI, 2.3–7.6; Table 5 and Supplemental Figure 4). The median RFS was 7.3 months (95% CI, 2.7–16.4) among patients who were MRD negative (<10^−4^ detectable leukemic blasts) and 1.9 months (95% CI, 0.8–6.0) among those who were not MRD negative. The median OS for all 70 patients was 7.5 months (95% CI, 4.0–11.8) with a median follow-up of 23.8 months. The most frequent grade ⩾ 3 AEs were anemia (36.0%), thrombocytopenia (21.0%), febrile neutropenia (17.0%), and hypokalemia (17.0%). The incidence of grade ⩾ 3 neurologic AEs was 4.0% and the incidence of grade ⩾ 3 CRS was 6.0%.

**Table 5.** Median RFS and median OS in pediatric patients with R/R or MRD-positive B-cell ALL treated with blinatumomab from pivotal studies and retrospective analyses.

Reference	Study name /descriptor	No. patients treated with blinatumomab	No. patients with CR and/or MRD response^a^ after treatment with blinatumomab (%)	Median RFS^b^, months (95% CI)	Median OS, months (95% CI)	Key safety results
Pivotal study						
von Stackelberg *et al*.^49^	Study MT-103-205	Phase I part, 49; phase II part, 44; Patients who received RP2D, 70 (2 patients were Ph positive)	27 (39.0); 14 of 27 had CR + complete MRD response^c^	4.4 (2.3–7.6); Patients with complete MRD response^c^, 7.3 (2.7–16.4); patients without MRD response, 1.9 (0.8–6.0)	7.5 (4.0–11.8)	The most frequent grade ⩾ 3 AEs were anemia (36.0%), thrombocytopenia (21.0%), febrile neutropenia (17.0%), and hypokalemia (17.0%). The incidence of grade ⩾ 3 neurologic AEs was 4.0% and grade ⩾ 3 CRS was 6.0%
Clinical studies supportive of the pivotal study						
Locatelli *et al.*^50^	RIALTO	110 (5 patients were Ph positive)	69 had CR as best response (62.7); 57 were MRD responders; 12 were MRD non-responders	8.5 (4.4–NE); MRD responders^a,d^, 8.5 (4.4–NE); MRD non-responders^d^, 9.2 (1.1–13.2)	13.1 (10.2–21.3); MRD responders^a^, 21.3 (19.7–24.5); MRD non-responders, 14.1 (2.0–NE)	The most frequent grade ⩾ 3 AEs were cytopenia (29.1%), infection (18.2%), and pyrexia (13.6%). The incidence of grade ⩾ 3 neurologic AEs was 5.5% and the incidence of grade ⩾ 3 CRS was 1.8%
Brown *et al.*^51^	AALL 1331	105	MRD-negativity rate was 75.0% after the first cycle, 66.0% after the second cycle of blinatumomab	Not reported, 2-year estimate was 54.4%	Not reported, 2-year estimate was 71.3%	The most frequent grade ⩾ 3 TEAEs were neutropenia (47.0%), lymphopenia (40.0%), and leukopenia (34.0%); the incidence of grade ⩾ 3 CRS was 1.0%, the incidence of grade ⩾ 3 encephalopathy was 4.0%, and incidence of grade ⩾ 3 seizure was 1.0%
Locatelli *et al.*^52^	Study 215	54	MRD-negativity rate was 90.0% 93.0% were MRD negative in patients who were MRD positive prior to blinatumomab	Not reported^e^	Not reported^f^	The most frequent grade ⩾ 3 TEAEs were thrombocytopenia (18.5%), stomatitis (18.5%), and neutropenia (16.7%). The incidence of grade ⩾ 3 neurologic AEs was 5.6% and the incidence of grade ⩾ 3 CRS was 0%
Long-term follow-up studies						
Gore *et al.*^53^	LTFU for MT-103-205	70	27^b^ (39.0) 16 of 27 had CR + complete MRD response^c^	Not reported	7.5 (4.0–11.8); patients with prior alloHSCT, 10.6 months (4.2–17.3); patients without prior alloHSCT, 4.3 (2.9–10.4) Complete MRD responders^c^, 14.6 months; MRD non-responders, 5.7 months	Not reported
Locatelli *et al.*^54^	LTFU for RIALTO	110	57 (51.8)	8.5 (4.7–14.0); MRD responders^a^, 8.0 (3.4–10.1); MRD non-responders, 2.8 (0.3–9.2) for	14.6 (11.0–NE); MRD responders^a^, NE; MRD non-responders, 9.3 (5.2–14.6)	The most frequent grade ⩾ 3 AEs were pyrexia (13.6%), a decrease in platelet count (10.0%), and febrile neutropenia (9.1%). Grade ⩾ 3 CRS was 1.8%; grade ⩾ 3 neurologic AEs (0%)
Locatelli *et al.*^55^	LTFU for Study 215	54	91.0% of patients who were MRD-positive^g^ prior to treatment with blinatumomab were MRD negative	Not reported^h^	Not reported^i^	Grade ⩾ 3 neurologic AEs, 5.6%; grade ⩾ 3 CRS, 0%
Retrospective analysis						
Queudeville *et al.*^56^	Single-center retrospective analysis (University Children’s Hospital Tübingen)	38 (2 patients were Ph positive)	11 (28.9)	6.2 (0–18.0)	11.1 (range, 0.2–113)	Grade ⩾ 3 CRS, 18.4%; grade ⩾ 3 neurologic AEs, 0%

a MRD response was defined as <10^−4^ detectable blasts.

b Median RFS was calculated in patients with CR. CR is defined as ⩽5% bone marrow and no evidence of disease.

c Complete MRD response was defined as no detectable blasts.

d *n* = 57 (46 patients with CR/CRh/CRi and an MRD response + 11 patients who had a CR at baseline and MRD response after blinatumomab).

e Incidence of events in the blinatumomab *versus* chemotherapy groups was 31.0% *versus* 57.0% [log-rank *p* < 0.001; HR, 0.33 (95% CI, 0.18–0.61)].

f The HR for OS was 0.43 (95% CI, 0.18–1.01).

g MRD positive was defined as ⩾10^−3^ detectable blasts.

h The HR for RFS for blinatumomab *versus* chemotherapy was 0.35 (95% CI, 0.20–0.61).

i The HR for OS was 0.34 (95% CI, 0.17–0.69).

AE, adverse event; ALL, acute lymphoblastic leukemia; alloHSCT, allogeneic hematopoietic stem cell transplantation; CI, confidence interval; CR, complete remission with full hematologic recovery; CRh, complete remission with partial hematologic recovery; CRi, complete remission with incomplete hematologic recovery; CRS, cytokine release syndrome; HR, hazard ratio; LTFU, long-term follow-up; MRD, measurable residual disease; NE, not estimable; NR, not reported; OS, overall survival; Ph, Philadelphia chromosome; RFS, relapse-free survival; RP2D, recommended phase II dose; R/R, relapsed refractory; TEAEs, treatment-emergent adverse events.

A long-term follow-up analysis assessed survival in 70 patients treated with the recommended phase II dose over 24 months from the start of blinatumomab.^53^ The median OS was 7.5 months (95% CI, 4.0–11.8). Prior alloHSCT was associated with prolonged survival after treatment with blinatumomab; the median OS was 10.6 months (95% CI, 4.2–17.3) for patients who had previously received alloHSCT *versus* 4.3 months (95% CI, 2.9–10.4) for those who had not (*p* = 0.1414). A total of 25 of 70 patients received alloHSCT following treatment with blinatumomab. OS appeared to be longer in patients who received alloHSCT as compared with those who did not. Thus, alloHSCT before or after treatment with blinatumomab improved the probability of OS. Patients with a complete MRD response (no target amplification with a minimum sensitivity of 10^−4^) had a median OS of 14.6 months compared with a median OS of 5.7 months in patients without a complete MRD response; this finding is similar to the trend seen in adult patients.

### Clinical study supportive of the pivotal study

Locatelli *et al*. reported results of an open-label, single-arm, expanded access international study in pediatric patients with CD19-positive R/R B-cell ALL (⩾5% blasts) or MRD-positive (<5% blasts but with MRD level ⩾10^−3^) B-cell ALL – RIALTO.^50^ Blinatumomab was administered at a dose of 5–15 µg/m^2^ per day as a 6-week induction cycle which comprised of cIV for 4 weeks, followed by a 2-week treatment-free period for a maximum of two induction cycles. Patients who achieved CR were eligible for three additional consolidation cycles. Of the 110 patients enrolled, 98 had R/R disease and 12 were MRD positive (⩽5% blasts at baseline). Of the 98 patients with R/R disease, 58 (59.2%) achieved CR and 11 of 12 MRD-positive patients (91.7%) became MRD negative at the end of the first two cycles of blinatumomab treatment. With a median follow-up of 11.2 months, the median RFS was 8.5 months (95% CI, 4.4–NE) in patients who achieved CR (*n* = 69). With a median follow-up of 17.4 months, the median OS for all patients (*n* = 110) was 13.1 months (95% CI, 10.2–21.3). Among MRD responders (*n* = 57, including patients who had a CR and were MRD negative in response to blinatumomab), the median RFS was 8.5 months (95% CI, 4.4–NE) and the median OS was 21.3 months (95% CI, 19.7–24.5) compared with a median RFS of 9.2 months (95% CI, 1.1–13.2) and a median OS of 14.1 months (95% CI, 2.0–NE) in MRD non-responders. The median RFS and median OS in this study were numerically longer compared with those reported in MT-103-205 by von Stackelberg *et al.*^49^ likely due to the enrollment of a higher number of patients with a lower tumor burden (<50% blasts) in RIALTO.

The effect of treatment with blinatumomab in pediatric patients with first-relapse B-cell ALL was investigated in two recent randomized phase III studies. In the first study, Brown *et al.* investigated the effects of blinatumomab *versus* chemotherapy when administered as consolidation therapy post-reinduction in children and young adults with first-relapse B-cell ALL.^51^ All patients received 4 weeks of reinduction chemotherapy followed by randomization to receive either two cycles of blinatumomab or two cycles of chemotherapy followed by alloHSCT. Of the 208 patients who were randomized, 118 (57%) received treatment with blinatumomab or chemotherapy. Randomization was terminated at the recommendation of the data and safety monitoring committee without compliance with the rules for termination due to concerns regarding clinical equipoise between the randomized treatments. With a median follow-up of 2.9 years, the 2-year estimate of disease-free survival was 54.4% for patients in the blinatumomab group *versus* 39.0% for those in the chemotherapy group [HR for disease progression or mortality, 0.07 (95% CI, 0.47–1.03); one-sided *p* = 0.03]. Among patients who received the randomized therapy, the 2-year estimate for OS was 71.3% for patients in the blinatumomab group *versus* 58.4% for those in the chemotherapy group [HR for mortality, 0.62 (95% CI, 0.39–0.98); one-sided *p* = 0.02]. Furthermore, after the first cycle of therapy with blinatumomab or chemotherapy, the rate of MRD negativity was greater with blinatumomab (75.0%) than with chemotherapy [32.0%; difference, 43.0% (95% CI, 31–55); *p* < 0.001], which persisted following the second cycle of blinatumomab or chemotherapy. Post-reinduction treatment with blinatumomab did not result in a statistically significant difference in RFS; however, the differences in OS and MRD negativity were significantly better with blinatumomab compared with chemotherapy.

A randomized phase III study by Locatelli *et al.* enrolled 108 pediatric patients older than 28 days and younger than 18 years with high-risk, first-relapse B-cell ALL with M1 (marrow blasts <5%) or M2 (marrow blasts ⩾5% but <25%) following treatment with standard-of-care induction and the first two cycles of consolidation chemotherapy.^52^ Patients were randomized to receive either one cycle of blinatumomab (15 µg/m^2^/day for 4 weeks) or chemotherapy for the third consolidation course before alloHSCT. With a median follow-up of 22.4 months, the RFS with blinatumomab was significantly better compared with chemotherapy [incidence of relapse, 31.5% *versus* 57.4%; HR, 0.33 (95% CI, 0.18–0.61); log-rank *p* < 0.01].^52^ A numerically higher percentage of patients treated with blinatumomab proceeded to alloHSCT (88.9%) compared with those treated with chemotherapy (70.4%) while in second continuous CR. The long-term follow-up over a median duration of 44.0 months confirmed that the RFS and OS were significantly improved with blinatumomab *versus* chemotherapy [HR for RFS was 0.35 (95% CI, 0.20–0.61, stratified log-rank *p* < 0.001) and HR for OS was 0.34 (95% CI, 0.17–0.69), stratified log-rank *p* = 0.002].^55^

### Key retrospective analyses or country-specific/ethnicity-specific studies

A retrospective evaluation^56^ of survival outcomes over a period of 10 years was reported for 38 patients, 16 of whom were part of the pivotal phase I/II study by von Stackelberg *et al.*^49^ A total of 13 of 38 patients (34.0%) achieved CR by the end of two cycles of treatment with blinatumomab. In these patients, over a median follow-up of 54.0 months, the median RFS was 6.2 months (95% CI, 0.0–18.0). The median OS for all patients treated with blinatumomab was 11.1 months (range, 0.2–113), which was numerically longer than the median OS reported in the pivotal study.^49^ Of the 13 patients with CR, only those who received alloHSCT after blinatumomab survived. Thus, alloHSCT after blinatumomab was critical for obtaining better OS in the long term. During the first cycle of blinatumomab, grade ⩾ 3 CRS was reported in 18.4% of patients and grade ⩾ 3 neurotoxicity was not reported in any patient treated with blinatumomab. No country-specific/ethnicity-specific studies in pediatric patients with R/R B-cell ALL have been reported.

### Key studies based on RWE in pediatric patients with R/R or MRD-positive B-cell ALL

A team from Italy reported the results of a retrospective observational study from 13 pediatric patients with R/R or MRD-positive B-cell ALL treated with blinatumomab (Table 6).^57^ The median RFS was 33.4 months (95% CI, 7.5–59.3) and the median OS was NR over a median follow-up of 16.0 months. In another real-world analysis, 8 of 15 Spanish patients achieved CR and were MRD negative after one cycle of blinatumomab; the median RFS and median OS were 8.5 months (range, 0–17) and 22.0 months (range, 3–41), respectively.^58^ As part of the NEUF study, Locatelli *et al.* reported on the results of a real-world analysis of 113 patients with B-cell ALL treated with blinatumomab of whom 72 had Ph-negative disease and 41 were MRD positive (Table 6).^59^ Upon treatment with two cycles of blinatumomab, 38 of 72 (53.0%) patients with R/R Ph-negative B-cell ALL achieved CR/CRh/CRi and 24 of 33 evaluable patients (72.7%) achieved an MRD response. The median RFS in patients with R/R Ph-negative B-cell ALL who had a CR/CRh/CRi was 5.4 months (95% CI, 3.9–NE) and the median OS for all patients with R/R Ph-negative B-cell ALL treated with blinatumomab was 8.2 months (95% CI, 5.8–18.2), which were comparable with the median RFS (4.4 months) and median OS (7.5 months) reported in the pivotal MT-103-205 study.^49^ Survival in patients with R/R Ph-negative B-cell ALL at 1 year after alloHSCT was 76.0% (95% CI, 51.3–89.3). Among MRD-positive patients, the median OS was NR over a median follow-up of 12.5 months. Upon additional censoring at the time of alloHSCT, the median OS in these patients was 19.3 months (95% CI, 6.9–19.3). Survival at 1 year after alloHSCT in these patients was 85% (95% CI, 49.8–96.1). A report based on RWE by Pawinska-Wasikowska *et al.* assessed the efficacy of blinatumomab in 13 Polish pediatric patients with R/R B-cell ALL.^60^ In all, 11 of the 13 patients received alloHSCT after treatment with blinatumomab, all of whom were alive without relapse with a follow-up of 25.4 months. These results support the idea that alloHSCT following treatment with blinatumomab could prolong survival in pediatric R/R B-cell ALL.

**Table 6.** Median RFS and median OS from real-world evidence studies in pediatric patients with R/R or MRD-positive B-cell ALL.

Reference and study name if applicable	Description of the patient population	No. patients treated with blinatumomab	No. patients with CR/CRh or MRD negative (%)	Median RFS^a^, months	Median OS, months	Key safety results
R/R B-cell ALL						
Locatelli *et al.*, NEUF study^61^	Patients from France, Italy, Russia, Spain, and the UK	72 with Ph-negative R/R B-cell ALL	38^b^ (52.8)	5.4 (IQR, 1.7–NE) for patients with Ph-negative R/R B-cell ALL; 4.9 upon censoring for alloHSCT; 5.4 months for patients with prior alloHSCT; 8.3 for patients without prior alloHSCT	8.2 (IQR, 4.6–NE) for patients with Ph-negative R/R B-cell ALL; 6.0 (IQR, 3.3–NE) upon censoring for alloHSCT, 6.4 for patients with prior alloHSCT; 9.2 for patients without prior alloHSCT	Not reported
Ampatzidou *et al.*^57^	Greek patients	9	6^b^ (66.7)	3.0 (range, 0.5–21.4)	8.7 (range, 1.4–47.1)	One patient developed grade 4 neurologic AE; no CRS
Fuster *et al.*^58^	Spanish patients	10	5 (50.0)	8.5 (range, 0–17.0)	22.0 (range, 3.0–41.0)	One patient developed grade 3 neurologic AE
Beneduce *et al.*^62^	Italian patients	13	6 (46.2)	33.4^c^ (7.5–59.3)	Not reported	The most frequent grade ⩾ 3 AEs were neurotoxicity (21.7) and hematologic toxicity (4.3%). No patient had grade ⩾ 3 CRS
Pawinska-Wasikowska *et al.*^60^	Polish patients	3	1 (33.3)	Not reported	Not reported	Not reported
MRD-positive^d^ B-cell ALL						
Locatelli *et al.*, NEUF study^61^	Patients from France, Italy, Russia, Spain, and the UK	41 (39 were Ph negative and two were Ph positive)	30 (73.1)	13.6 for MRD responders^e^; 9.1 upon censoring for alloHSCT	NR for MRD responders^e^; 19.3 (7.3–19.3) for MRD responders^e^ upon censoring for alloHSCT	Not reported
Ampatzidou *et al.*^57^	Greek patients	2	2 (100.0)	7.4 for patients with an MRD response^e^; 0.5 for patients without an MRD response^d^	7.6 for patients with MRD response^c^; 3.0 for patients without an MRD response^e^	Not reported
Fuster *et al.*^58^	Spanish patients	4	4 (100.0)	Not reported	Not reported	Not reported
Beneduce *et al.*^62^	Italian patients	26	19 (34.6)	Not reported	Not reported	Not reported
Pawinska-Wasikowska *et al.*^60^	Polish patients	10	9 (90.0)	Not reported	Not reported	Not reported

a Median RFS was calculated in patients with CR/CRh/CRi or MRD responders.

b Patients with CR/CRh/CRi. CR is defined as ⩽5% bone marrow and no evidence of disease, platelet count >100,000 per µL, and absolute neutrophil count >1000 per µL. CRh is defined as ⩽5% bone marrow blasts and no evidence of disease, platelet count >50,000 per µL, and absolute neutrophil count >500 per µL. CRi is defined as CRh was defined as ⩽5% bone marrow blasts and no evidence of disease, platelet count of >100,000 per microliter or absolute neutrophil count of >1000 per microliter.

c In patients with R/R or MRD-positive disease prior to treatment with blinatumomab.

d MRD-positive was defined as ⩾10^−3^ detectable blasts.

e MRD response was defined as <10^−4^ detectable blasts or ⩾2-log reduction of MRD value before and after blinatumomab administration.

AE, adverse event; ALL, acute lymphoblastic leukemia; alloHSCT, allogeneic hematopoietic stem cell transplantation; CI, confidence interval; CR, complete remission with full hematologic recovery; CRh, complete remission with partial hematologic recovery; CRi, complete remission with incomplete hematologic recovery; CRS, cytokine release syndrome; IQR, interquartile range; MRD, measurable residual disease; NE, not estimable; NR, not reached; OS, overall survival; Ph, Philadelphia chromosome; R/R, relapsed/refractory; RFS, relapse-free survival.

## Discussion

Results from pivotal studies, retrospective analyses based on clinical studies, studies in patients from specific countries and/or ethnicities, and the analysis of RWE have provided an opportunity to compare survival outcomes in specific subgroups of patients treated with blinatumomab. A key finding of this literature review was that, although treatment with blinatumomab was beneficial as both first and later salvage, the effects of treatment were more favorable when blinatumomab was administered as the first salvage therapy and in patients with molecularly resistant disease compared with those treated for overt recurrence, especially if associated with a bone marrow blast infiltration of greater than 50.0%.^63^ The median OS in patients with R/R Ph-negative B-cell ALL who received blinatumomab as first salvage was numerically longer compared with the median OS reported in the overall study population of pivotal studies (MT-103-211 and TOWER).^15–17,22,23^ In addition, the safety profile for patients who received blinatumomab as first salvage was generally similar to the safety profile for all patients from the MT-103-211 and TOWER studies.^14,15^

Patients who achieved a complete MRD response after treatment with blinatumomab had better RFS and OS compared with patients who did not achieve a CR or MRD response. The median RFS and median OS in patients with R/R Ph-negative B-cell ALL who achieved CR/CRh and a complete MRD response after blinatumomab therapy were numerically longer compared with the median RFS and median OS reported in patients from the pivotal MT-103-211 and TOWER studies without the incidence of additional AEs.^15,17,26^ Analysis of long-term outcomes in MRD-positive patients who achieved a complete MRD response after treatment with blinatumomab found a 5-year survival rate of 50.0% regardless of the status of alloHSCT.^39^ Patients with B-cell ALL who are MRD positive before alloHSCT have a significantly higher rate of relapse and lower OS compared with patients who are MRD negative.^64,65^ Thus, patients who are MRD positive before alloHSCT could be considered for treatment with blinatumomab maintenance after transplantation. In this regard, Gaballa *et al.* recently reported on the feasibility of four cycles of blinatumomab administered every 3 months during the first year after transplantation to prevent relapse in high-risk B-cell ALL patients.^66^ The median time from transplantation to the first cycle of blinatumomab was 78 days (range, 44–105). The treatment was well tolerated, and no relevant toxicity was recorded. Additional studies are needed to document if this approach can be of benefit for disease eradication.

The goal of therapy in R/R B-cell ALL is to induce CR followed by consolidation of CR with alloHSCT in most adult and selected pediatric patients. The role of alloHSCT in the extension of survival in patients who respond to blinatumomab *versus* those who do not respond to blinatumomab is not yet fully understood, which is due, in part, to the fact that neither are the indications for alloHSCT defined in prospective trials nor is alloHSCT a standardized procedure, with much heterogeneity in the use of conditioning regimens, donor selection, and management of post-transplant immune suppression across the large number of centers. This creates room for hard-to-characterize biases and makes conclusions difficult. A retrospective analysis in adults with R/R B-cell ALL showed that survival outcomes were dependent on response to treatment with blinatumomab irrespective of whether patients received alloHSCT following blinatumomab.^25^ By contrast, an analysis of long-term survival of patients from two clinical studies showed that treatment with blinatumomab followed by alloHSCT improved median OS.^20^ Additional studies designed to prospectively assess the effect of blinatumomab followed by alloHSCT in adults with R/R B-cell ALL are therefore needed. In the case of pediatric patients with R/R B-cell ALL, Queudeville *et al.*^56^ demonstrated a positive effect of blinatumomab followed by alloHSCT on OS and the recent RIALTO trial^50^ showed a trend toward better RFS and OS in patients treated with blinatumomab followed by alloHSCT.^50,56^ Results of the recently published randomized phase III study by Locatelli *et al.*,^52,55^ where alloHSCT was prospectively indicated in patients with high-risk first-relapse B-cell ALL following a third consolidation course with one cycle of blinatumomab, demonstrated a significant improvement in EFS and OS than in patients treated with chemotherapy.

Recent years have witnessed the introduction of other forms of immunotherapy in patients with B-cell ALL, including the anti-CD22 antibody–drug conjugate inotuzumab ozogamicin (InO) and anti-CD19 chimeric antigen receptor T-cell therapy (CART-19), which have shown significant efficacy in patients with R/R B-cell ALL.^67,68^ Results from different trials using blinatumomab, InO, or CART-19 are often compared; however, a better approach could be to design a strategy for the use of these agents in combination and/or sequentially with the intent to improve long-term survival. A recent case study demonstrated positive results in patients with Ph-positive R/R B-cell ALL who were treated sequentially with InO and blinatumomab.^69^ The IntReALL 2020 study protocol will test the sequential use of both InO and blinatumomab in children with the first relapse of B-cell ALL (Locatelli F, personal communication). Patients with prior exposure to blinatumomab have been demonstrated to respond to subsequent treatment with CART-19. In the pivotal phase I/II ZUMA-3 study, the rate of CR/CRi among patients with R/R B-cell ALL treated with CART-19 with prior exposure to blinatumomab in any line (12 of 21 patients; 57%) was similar to the rate of CR/CRi observed in the overall patient population treated with CART-19 (31 of 45 patients; 69%).^70^ Another report based on a retrospective multicenter analysis of 420 patients aged ⩽25 years who were treated with CART-19 demonstrated that although prior exposure to blinatumomab led to downregulation of CD19 expression, it did not preclude a response to salvage therapy with CART-19.^71^ Poor response to prior treatment with blinatumomab, rather than prior exposure to blinatumomab, was predictive of poor outcome to subsequent therapy with CART-19. This finding suggests that an intrinsic T-cell dysfunction is more relevant to predict the probability of response to CART-19 than prior exposure to a CD19-directed treatment.

We found that the median RFS and median OS reported in studies based on RWE in adult patients with R/R B-cell ALL treated with blinatumomab were longer compared with the median RFS and median OS reported in pivotal studies (MT-103-211, TOWER, and ALCANTARA). A possible explanation could be the enrollment of a greater number of heavily pretreated patients and patients with relapse after alloHSCT in the pivotal studies.

Novel treatment strategies could further improve survival outcomes in patients with R/R or MRD-positive B-cell ALL. The combination of blinatumomab with InO or other targeted therapies followed by alloHSCT and subsequent monitoring of MRD could help improve survival outcomes. A subcutaneous formulation of blinatumomab that allows for greater systemic exposure to blinatumomab compared with the dose used for intravenous administration is currently being evaluated and has demonstrated promising early signals.

## Conclusion

This review of the published literature demonstrated the consistency of the efficacy and safety of blinatumomab for the treatment of R/R B-cell ALL from pivotal studies as well as studies based on retrospective analyses and RWE. The results were consistently superior when blinatumomab was given in the first salvage compared with later salvages. While the comparative results of blinatumomab, InO, and CART-19 may be of interest, we believe such questions are not as critical as investigating the efficacy and safety of the combinations of these therapies - blinatumomab and InO and its combination with standard chemotherapy - and the value of CART-19 or alloHSCT after induction of CR and/or MRD-negative status. This should be the focus of our current and future studies in ALL.

## Supplemental Material

sj-docx-1-tah-10.1177_20406207231201454 – Supplemental material for Survival outcomes in patients with relapsed/refractory or MRD-positive B-cell acute lymphoblastic leukemia treated with blinatumomab Click here for additional data file. Supplemental material, sj-docx-1-tah-10.1177_20406207231201454 for Survival outcomes in patients with relapsed/refractory or MRD-positive B-cell acute lymphoblastic leukemia treated with blinatumomab by Hagop M. Kantarjian, Aaron C. Logan, Faraz Zaman, Nicola Gökbuget, Ralf C. Bargou, Yi Zeng, Gerhard Zugmaier and Franco Locatelli in Therapeutic Advances in Hematology

sj-tif-2-tah-10.1177_20406207231201454 – Supplemental material for Survival outcomes in patients with relapsed/refractory or MRD-positive B-cell acute lymphoblastic leukemia treated with blinatumomab Click here for additional data file. Supplemental material, sj-tif-2-tah-10.1177_20406207231201454 for Survival outcomes in patients with relapsed/refractory or MRD-positive B-cell acute lymphoblastic leukemia treated with blinatumomab by Hagop M. Kantarjian, Aaron C. Logan, Faraz Zaman, Nicola Gökbuget, Ralf C. Bargou, Yi Zeng, Gerhard Zugmaier and Franco Locatelli in Therapeutic Advances in Hematology

sj-tif-3-tah-10.1177_20406207231201454 – Supplemental material for Survival outcomes in patients with relapsed/refractory or MRD-positive B-cell acute lymphoblastic leukemia treated with blinatumomab Click here for additional data file. Supplemental material, sj-tif-3-tah-10.1177_20406207231201454 for Survival outcomes in patients with relapsed/refractory or MRD-positive B-cell acute lymphoblastic leukemia treated with blinatumomab by Hagop M. Kantarjian, Aaron C. Logan, Faraz Zaman, Nicola Gökbuget, Ralf C. Bargou, Yi Zeng, Gerhard Zugmaier and Franco Locatelli in Therapeutic Advances in Hematology

sj-tif-4-tah-10.1177_20406207231201454 – Supplemental material for Survival outcomes in patients with relapsed/refractory or MRD-positive B-cell acute lymphoblastic leukemia treated with blinatumomab Click here for additional data file. Supplemental material, sj-tif-4-tah-10.1177_20406207231201454 for Survival outcomes in patients with relapsed/refractory or MRD-positive B-cell acute lymphoblastic leukemia treated with blinatumomab by Hagop M. Kantarjian, Aaron C. Logan, Faraz Zaman, Nicola Gökbuget, Ralf C. Bargou, Yi Zeng, Gerhard Zugmaier and Franco Locatelli in Therapeutic Advances in Hematology

sj-tif-5-tah-10.1177_20406207231201454 – Supplemental material for Survival outcomes in patients with relapsed/refractory or MRD-positive B-cell acute lymphoblastic leukemia treated with blinatumomab Click here for additional data file. Supplemental material, sj-tif-5-tah-10.1177_20406207231201454 for Survival outcomes in patients with relapsed/refractory or MRD-positive B-cell acute lymphoblastic leukemia treated with blinatumomab by Hagop M. Kantarjian, Aaron C. Logan, Faraz Zaman, Nicola Gökbuget, Ralf C. Bargou, Yi Zeng, Gerhard Zugmaier and Franco Locatelli in Therapeutic Advances in Hematology

## Declarations

**Ethics approval and consent to participate:** Not applicable.

**Consent for publication:** Not applicable.

**Availability of data and materials:** Data sharing is not applicable to this article as no new data were created or analyzed in this study.
