# Supplementary material for: Survival outcomes in patients with relapsed/refractory or MRD-positive B-cell acute lymphoblastic leukemia treated with blinatumomab
Source: Ther Adv Hematol. 2023 Oct 9;14:20406207231201454. doi: 10.1177/20406207231201454 (PMC10563488; doi:10.1177/20406207231201454)
Supplement: sj-docx-1-tah-10.1177_20406207231201454 – Supplemental material for Survival outcomes in patients with relapsed/refractory or MRD-positive B-cell acute lymphoblastic leukemia treated with blinatumomab [file sj-docx-1-tah-10.1177_20406207231201454.docx]

**Supplementary Figure Legends**

**Supplementary Figure 1. Median RFS and OS in adult patients with Ph-negative R/R B-cell ALL treated with blinatumomab from pivotal studies, retrospective analyses, single country or ethnicity-specific studies, and studies based on real-world evidence.**

^a^In patients with CR/CRh or CR/CRh/CRi. CR is defined as ≤5% bone marrow and no evidence of disease, platelet count > 100,000 per µL, and absolute neutrophil count > 1000 per µL. CRh is defined as ≤5% bone marrow blasts and no evidence of disease, platelet count > 50,000 per µL, and absolute neutrophil count > 500 per µL. CRi is defined as CRh was defined as ≤5% bone marrow blasts and no evidence of disease, platelet count of > 100,000 per microliter or absolute neutrophil count of > 1000 per microliter.

^b^N=21 (2 patients were Ph-positive, the number of Ph-negative patients were not reported).

^c^N=227 (166 patients were Ph-negative).

^d^N=166 (all patients were Ph-negative).

^e^N=38 (11 patients were Ph-positive, the number of Ph-negative patients was not reported).

^f^N=106 (all patients were Ph-negative).

MRD-response is defined as < 10^-4^ detectable leukemic blasts with the use of allele-specific real-time quantitative PCR for clonal rearrangements of immunoglobulin or T-cell receptor genes (assay sensitivity of ≤ 10^-4^ detectable blasts).

ALL, acute lymphoblastic leukemia; alloHSCT, allogenic hematopoietic stem cell transplant; CR, complete remission with full hematologic recovery; CRh, complete remission with partial hematologic recovery; CRi, complete remission with incomplete hematologic recovery; LTFU, long-term follow-up; MRD, measurable residual disease; OS, overall survival; R/R, relapsed/refractory; Ph, Philadelphia chromosome; RFS, relapse-free survival.

**Supplementary Figure 2. Median RFS and OS in adult patients with Ph-positive R/R B-cell ALL treated with blinatumomab from pivotal studies, retrospective analyses, and studies based on real-world evidence**

^a^In patients with CR/CRh. CR is defined as ≤5% bone marrow and no evidence of disease, platelet count > 100,000 per µL, and absolute neutrophil count > 1000 per µL. CRh is defined as ≤5% bone marrow blasts and no evidence of disease, platelet count > 50,000 per µL, and absolute neutrophil count > 500 per µL.

^b^Patients with CR/CRh + complete MRD response. Complete MRD response is defined as no

detectable PCR amplification of *BCR-ABL1* genes (sensitivity ≥ 10^-5^).

^c^N=32, patients were treated with blinatumomab alone.

^d^N=34, all patients were Ph-positive.

ALL, acute lymphoblastic leukemia; CR, complete remission with full hematologic recovery; CRh, complete remission with partial hematologic recovery; LTFU, long-term follow-up; MRD, measurable residual disease; OS, overall survival; Ph, Philadelphia chromosome; R/R relapsed/refractory; RFS, relapse-free survival.

**Supplementary Figure 3. Median RFS and OS in adult patients with MRD-positive B-cell ALL treated with blinatumomab from pivotal studies and studies based on real-world evidence.**

^a^At 18 months after initiation of blinatumomab in patients with CR.

^b^MRD-positive is defined as presence of ≥ 10^−3^ leukemic blasts.

^c^All patients in the analysis set were Ph-negative.

^d^Complete MRD response is defined as no target amplification with a minimum sensitivity of 10^-4^.

^e^83/109 patients were Ph-negative and 26/109 patients were Ph-positive.

^f^6/12 patients were Ph-positive.

ALL, acute lymphoblastic leukemia; CR, complete remission with full hematologic recovery; MRD, measurable residual disease; OS, overall survival; Ph, Philadelphia Chromosome; RFS, relapse-free survival.

**Supplementary Figure 4. Median RFS and OS for pediatric patients with R/R or MRD-positive B-cell ALL treated with blinatumomab from pivotal studies, retrospective analyses, single country or ethnicity-specific studies, and studies based on real-world evidence.**

^a^Calculated in patients with CR/CRh. CR is defined as ≤5% bone marrow and no evidence of disease, platelet count > 100,000 per µL, and absolute neutrophil count > 1000 per µL. CRh is defined as ≤5% bone marrow blasts and no evidence of disease, platelet count > 50,000 per µL, and absolute neutrophil count > 500 per µL.

^b^Complete MRD response is defined as no target amplification with a minimum sensitivity of 10^-4^.

^c^MRD-response is defined as < 10^-4^ detectable leukemic blasts with the use of allele-specific real-time quantitative PCR for clonal rearrangements of immunoglobulin or T-cell receptor genes (assay sensitivity of ≤ 10^-4^ detectable blasts).

^d^In patients with R/R or CR + MRD-positive disease at baseline. MRD-positive is defined as presence of ≥ 10^−3^ leukemic blasts.

ALL, acute lymphoblastic leukemia; alloHSCT, allogenic hematopoietic stem cell transplant; CR, complete remission; LTFU, long-term follow-up; MRD, measurable residual disease; OS, overall survival; Ph, Philadelphia chromosome; R/R, relapsed/refractory; RFS, relapse-free survival.

**References**

1. Topp MS, Gökbuget N, Stein AS, et al. Safety and activity of blinatumomab for adult patients with relapsed or refractory B-precursor acute lymphoblastic leukaemia: a multicentre, single-arm, phase 2 study. *The Lancet Oncology.* 2015;16(1):57-66.

2. Kantarjian H, Stein A, Gökbuget N, et al. Blinatumomab versus chemotherapy for advanced acute lymphoblastic leukemia. *The New England journal of medicine.* 2017;376(9):836-847.

3. Topp MS, Gökbuget N, Zugmaier G, et al. Phase II trial of the anti-CD19 bispecific T cell-engager blinatumomab shows hematologic and molecular remissions in patients with relapsed or refractory B-precursor acute lymphoblastic leukemia. *Journal of clinical oncology : official journal of the American Society of Clinical Oncology.* 2014;32(36):4134-4140.

4. Aboudalle I, Kantarjian HM, Short NJ, et al. Long term follow-up on phase 2 study on the efficacy and safety of blinatumomab in adult patients with relapsed refractory B-precursor acute lymphoblastic leukemia. Abstract accepted for presentation at 60th Annual Meeting of the American-Society-of-Hematology. *Blood.* 2018;132:4017.

5. Topp M, Stein AS, Zugmaier G, et al. Long-term survival of adults with B-cell precursor acute lymphoblastic leukemia after treatment with blinatumomab and subsequent allogeneic hematopoietic stem cell transplantation. Poster presented at the American Society of Clinical Oncology (ASCO) Annual Meeting; Chicago, IL; June 1-5, 2018. *Journal of Clinical Oncology.* 2018;36(15_suppl):7044-7044.

6. Topp MS, Gökbuget N, Zugmaier G, et al. Long-term survival of patients with relapsed/refractory acute lymphoblastic leukemia treated with blinatumomab. *Cancer.* 2021;127(4):554-559.

7. Zugmaier G, Gökbuget N, Klinger M, et al. Long-term survival and T-cell kinetics in relapsed/refractory ALL patients who achieved MRD response after blinatumomab treatment. *Blood.* 2015;126(24):2578-2584.

8. Stein AS, Kantarjian H, Gökbuget N, et al. Blinatumomab for acute lymphoblastic leukemia relapse after allogeneic hematopoietic stem cell transplantation. *Biology of blood and marrow transplantation : journal of the American Society for Blood and Marrow Transplantation.* 2019;25(8):1498-1504.

9. Dombret H, Topp MS, Schuh AC, et al. Blinatumomab versus chemotherapy in first salvage or in later salvage for B-cell precursor acute lymphoblastic leukemia. *Leukemia & lymphoma.* 2019;60(9):2214-2222.

10. Topp MS, Stein AS, Gökbuget N, et al. Blinatumomab as first salvage versus second or later salvage in adults with relapsed/refractory B-cell precursor acute lymphoblastic leukemia: Results of a pooled analysis. *Cancer medicine.* 2021;10(8):2601-2610.

11. Rambaldi A, Huguet F, Zak P, et al. Blinatumomab consolidation and maintenance therapy in adults with relapsed/refractory B-precursor acute lymphoblastic leukemia. *Blood advances.* 2020;4(7):1518-1525.

12. Jabbour E, Short NJ, Jorgensen JL, et al. Differential impact of minimal residual disease negativity according to the salvage status in patients with relapsed/refractory B-cell acute lymphoblastic leukemia. *Cancer.* 2017;123(2):294-302.

13. Gökbuget N, Kantarjian HM, Brüggemann M, et al. Molecular response with blinatumomab in relapsed/refractory B-cell precursor acute lymphoblastic leukemia. *Blood advances.* 2019;3(20):3033-3037.

14. Kantarjian HM, Stein AS, Bargou RC, et al. Blinatumomab treatment of older adults with relapsed/refractory B-precursor acute lymphoblastic leukemia: Results from 2 phase 2 studies. *Cancer.* 2016;122(14):2178-2185.

15. Aldoss I, Song J, Stiller T, et al. Correlates of resistance and relapse during blinatumomab therapy for relapsed/refractory acute lymphoblastic leukemia. *American journal of hematology.* 2017;92(9):858-865.

16. Yoon JH, Min GJ, Park SS, et al. Feasible outcome of blinatumomab followed by allogeneic hematopoietic cell transplantation for adults with Philadelphia chromosome-negative acute lymphoblastic leukemia in first salvage. *Cancer medicine.* 2019;8(18):7650-7659.

17. Zhou H, Yin Q, Jin J, et al. Efficacy and safety of blinatumomab in Chinese adults with Ph-negative relapsed/refractory B-cell precursor acute lymphoblastic leukemia: A multicenter open-label single-arm China registrational study. *Hematology (Amsterdam, Netherlands).* 2022;27(1):917-927.

18. Jung S-H, Lee S-R, Yang D-H, et al. Efficacy and safety of blinatumomab treatment in adult Korean patients with relapsed/refractory acute lymphoblastic leukemia on behalf of the Korean Society of Hematology ALL Working Party. *Annals of hematology.* 2019;98(1):151-158.

19. Kiyoi H, Morris JD, Oh I, et al. Phase 1b/2 study of blinatumomab in Japanese adults with relapsed/refractory acute lymphoblastic leukemia. *Cancer science.* 2020;111(4):1314-1323.

20. Kobayashi Y, Oh I, Miyamoto T, et al. Efficacy and safety of blinatumomab: Post hoc pooled analysis in Asian adults with relapsed/refractory B-cell precursor acute lymphoblastic leukemia. *Asia-Pacific journal of clinical oncology.* 2021;18(3):8.

21. Apel A, Ofran Y, Wolach O, et al. Safety and efficacy of blinatumomab: a real world data. *Annals of hematology.* 2020;99(4):835-838.

22. Badar T, Szabo A, Advani A, et al. Real-world outcomes of adult B-cell acute lymphocytic leukemia patients treated with blinatumomab. *Blood advances.* 2020;4(10):2308-2316.

23. Badar T, Szabo A, Dinner S, et al. Sequencing of novel agents in relapsed/refractory B-cell acute lymphoblastic leukemia: blinatumomab and inotuzumab ozogamicin may have comparable efficacy as first or second novel agent therapy in relapsed/refractory acute lymphoblastic leukemia. *Cancer.* 2021;127(7):1039-1048.

24. Cabannes-Hamy A, Brissot E, Leguay T, et al. Tumor burden and outcome after blinatumomab in adult B-cell acute lymphoblastic leukemia. Results of the French-cyto study. *European Hematology Association.* 2020;294311; EP392.

25. Boissel N, Ribera J-M, Chiaretti S, et al. Treatment of adults with relapsed/refractory Philadelphia chromosome negative acute lymphoblastic leukemia with blinatumomab in a real-world setting: results from the NEUF study. Poster presented at the 61st American Society of Hematology (ASH) Annual Meeting; Orlando, Florida; December 7–10, 2019. *Blood.* 2019;134(Supplement_1):2627-2627.

26. Martinelli G, Boissel N, Chevallier P, et al. Complete hematologic and molecular response in adult patients with relapsed/refractory philadelphia chromosome-positive B-precursor acute lymphoblastic leukemia following treatment with blinatumomab: results from a phase II, single-arm, multicenter study. *Journal of clinical oncology : official journal of the American Society of Clinical Oncology.* 2017;35(16):1795-1802.

27. Martinelli G, Boissel N, Chevallier P, et al. Long-term follow-up of blinatumomab in patients with relapsed/refractory Philadelphia chromosome-positive B-cell precursor acute lymphoblastic leukaemia: Final analysis of ALCANTARA study. *European journal of cancer (Oxford, England : 1990).* 2021;146:107-114.

28. Chiaretti S PC, Ribera JM, Bassan R, Rambaldi A, Sokolov AN, Foa R, Alam N, Pezzani I, Brescianini A, Kreuzbauer G, Boissel N. Treatment of adults with relapsed/refractory Philadelphia chromosome-positive acute lymphoblastic leukemia with blinatumomab in a real-world setting: results from the NEUF study. Poster presented at the Virtual 25th Congress of European Haematology Association (EHA); June 11–14, 2020. *EHA.* 2020.

29. Gökbuget N, Dombret H, Bonifacio M, et al. Blinatumomab for minimal residual disease in adults with B-cell precursor acute lymphoblastic leukemia. *Blood.* 2018;131(14):1522-1531.

30. Gökbuget N, Dombret H, Giebel S, et al. Blinatumomab vs historic standard-of-care treatment for minimal residual disease in adults with B-cell precursor acute lymphoblastic leukaemia. *European journal of haematology.* 2020;104(4):299-309.

31. Boissel N, Bassan R, Ribera J-M, et al. Treatment of adults with minimal residual disease (MRD) positive acute lymphoblastic leukemia with blinatumomab in a real-world setting: results from the NEUF study. Poster presented at the 61st American Society of Hematology (ASH) Annual Meeting; Orlando, Florida; December 7–10, 2019. *Blood.* 2019;134(Supplement_1):2624-2624.

32. von Stackelberg A, Locatelli F, Zugmaier G, et al. Phase I/Phase II study of blinatumomab in pediatric patients with relapsed/refractory acute lymphoblastic leukemia. *Journal of clinical oncology : official journal of the American Society of Clinical Oncology.* 2016;34(36):4381-4389.

33. Gore L, Locatelli F, Zugmaier G, et al. Survival after blinatumomab treatment in pediatric patients with relapsed/refractory B-cell precursor acute lymphoblastic leukemia. *Blood cancer journal.* 2018;8(9):80.

34. Locatelli F, Zugmaier G, Mergen N, et al. Blinatumomab in pediatric patients with relapsed/refractory acute lymphoblastic leukemia: results of the RIALTO trial, an expanded access study. *Blood cancer journal.* 2020;10(7):77.

35. Locatelli F, Zugmaier G, Bader P, et al. Blinatumomab in pediatric relapsed/refractory B-cell precursor acute lymphoblastic leukemia: RIALTO expanded access study final analysis. *Blood advances.* 2022;6(3):1004–1014.

36. Queudeville M, Schlegel P, Heinz AT, et al. Blinatumomab in pediatric patients with relapsed/refractory B-cell precursor acute lymphoblastic leukemia. *European journal of haematology.* 2021;106(4):473-483.

37. Ampatzidou M, Kattamis A, Baka M, et al. Insights from the Greek experience of the use of Blinatumomab in pediatric relapsed and refractory acute lymphoblastic leukemia patients. *Neoplasma.* 2020;67(6):1424-1430.

38. Fuster JL, Molinos-Quintana A, Fuentes C, et al. Blinatumomab and inotuzumab for B cell precursor acute lymphoblastic leukaemia in children: a retrospective study from the Leukemia Working Group of the Spanish Society of Pediatric Hematology and Oncology (SEHOP). *British journal of haematology.* 2020;190(5):764-771.

39. Beneduce G, De Matteo A, Stellato P, et al. Blinatumomab in Children and Adolescents with Relapsed/Refractory B Cell Precursor Acute Lymphoblastic Leukemia: A Real-Life Multicenter Retrospective Study in Seven AIEOP (Associazione Italiana di Ematologia e Oncologia Pediatrica) Centers. *Cancers.* 2022;14(2).

40. Locatelli F, Maschan A, Boissel N, et al. Pediatric patients with acute lymphoblastic leukemia treated with blinatumomab in a real-world setting: Results from the NEUF study. *Pediatric blood & cancer.* 2022;69(4):e29562.
