# Supplementary figures and images for: Survival outcomes in patients with relapsed/refractory or MRD-positive B-cell acute lymphoblastic leukemia treated with blinatumomab
Source: Ther Adv Hematol. 2023 Oct 9;14:20406207231201454. doi: 10.1177/20406207231201454 (PMC10563488; doi:10.1177/20406207231201454)

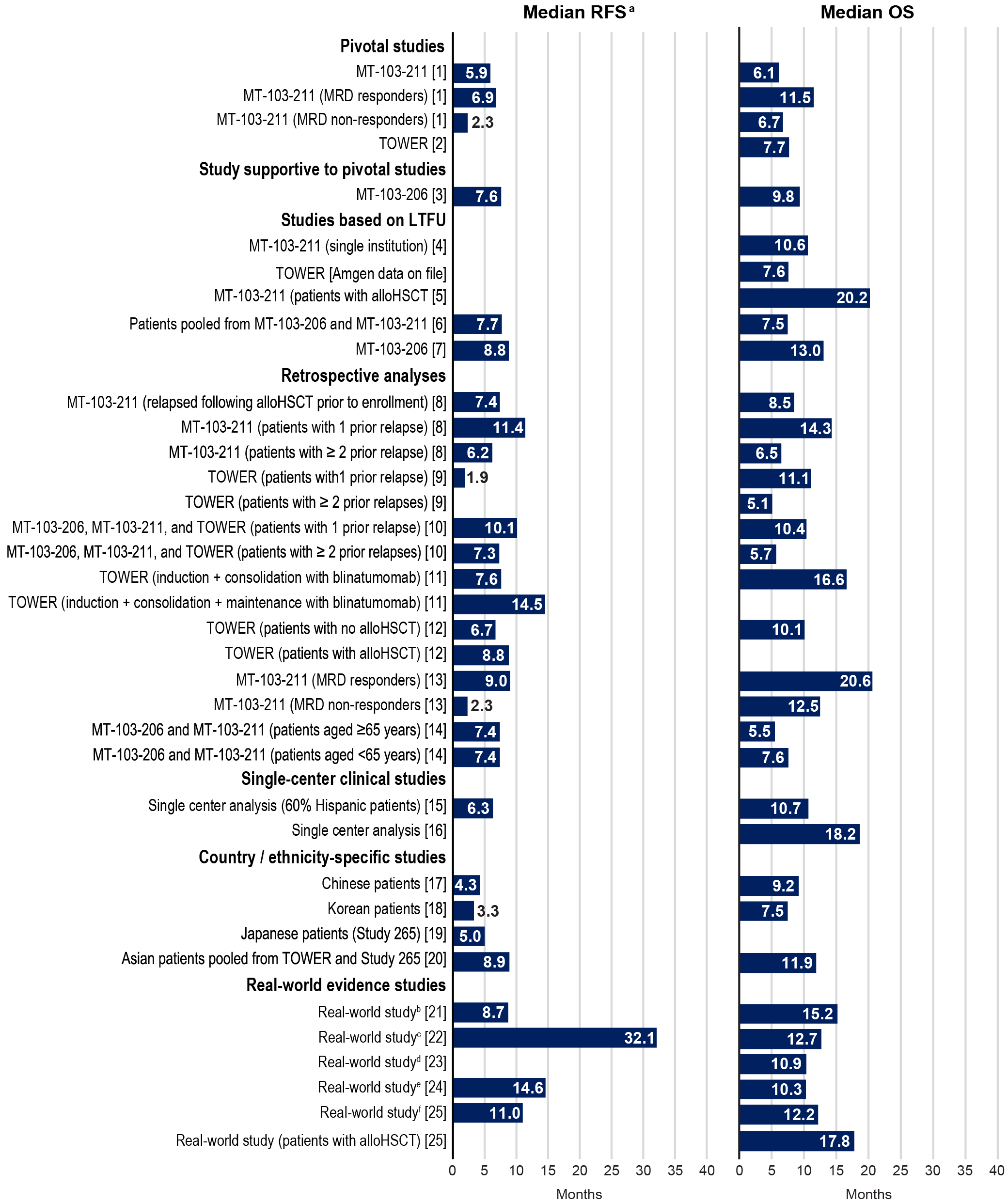

Supplement: sj-tif-2-tah-10.1177_20406207231201454 – Supplemental material for Survival outcomes in patients with relapsed/refractory or MRD-positive B-cell acute lymphoblastic leukemia treated with blinatumomab [file sj-tif-2-tah-10.1177_20406207231201454.tif]

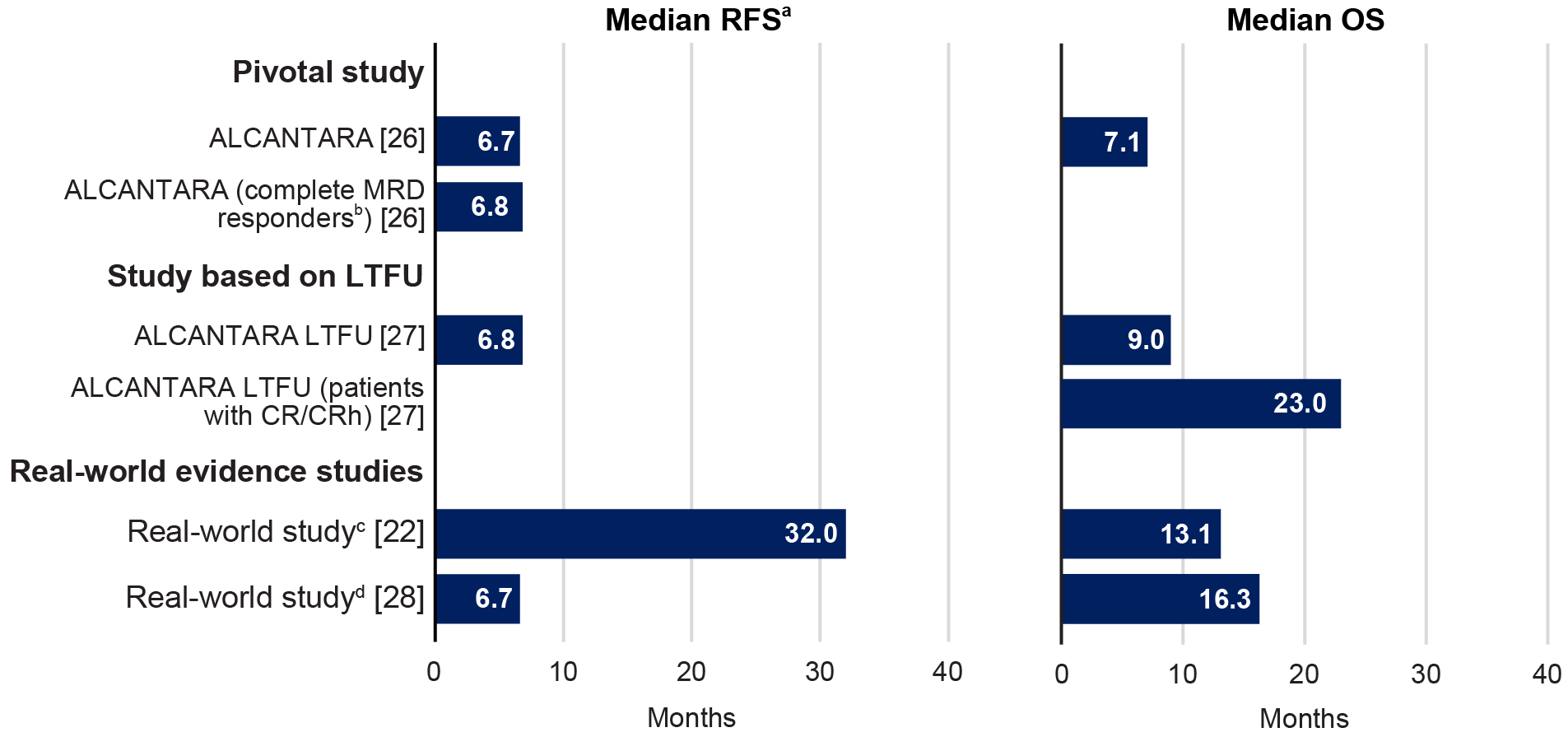

Supplement: sj-tif-3-tah-10.1177_20406207231201454 – Supplemental material for Survival outcomes in patients with relapsed/refractory or MRD-positive B-cell acute lymphoblastic leukemia treated with blinatumomab [file sj-tif-3-tah-10.1177_20406207231201454.tif]

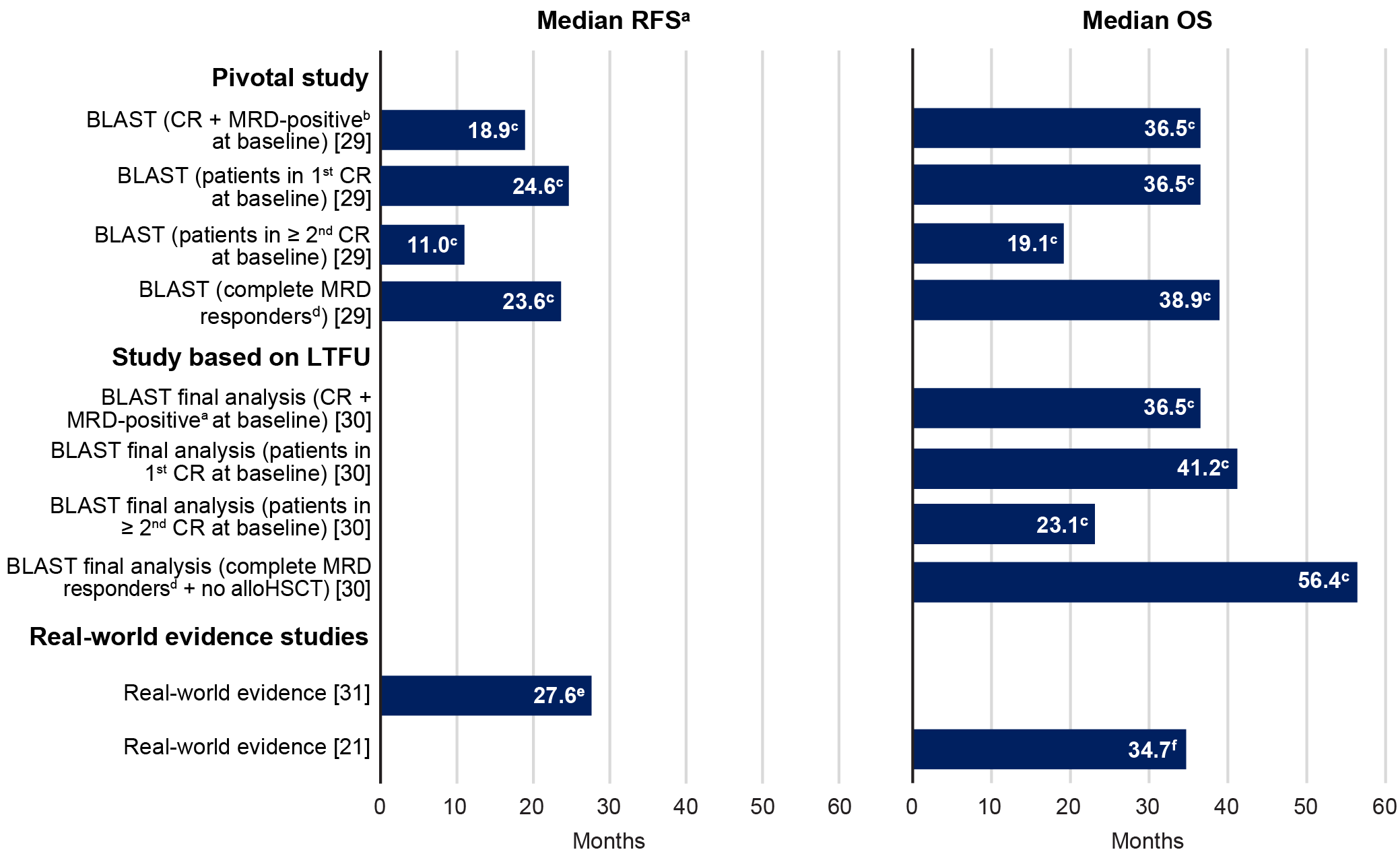

Supplement: sj-tif-4-tah-10.1177_20406207231201454 – Supplemental material for Survival outcomes in patients with relapsed/refractory or MRD-positive B-cell acute lymphoblastic leukemia treated with blinatumomab [file sj-tif-4-tah-10.1177_20406207231201454.tif]

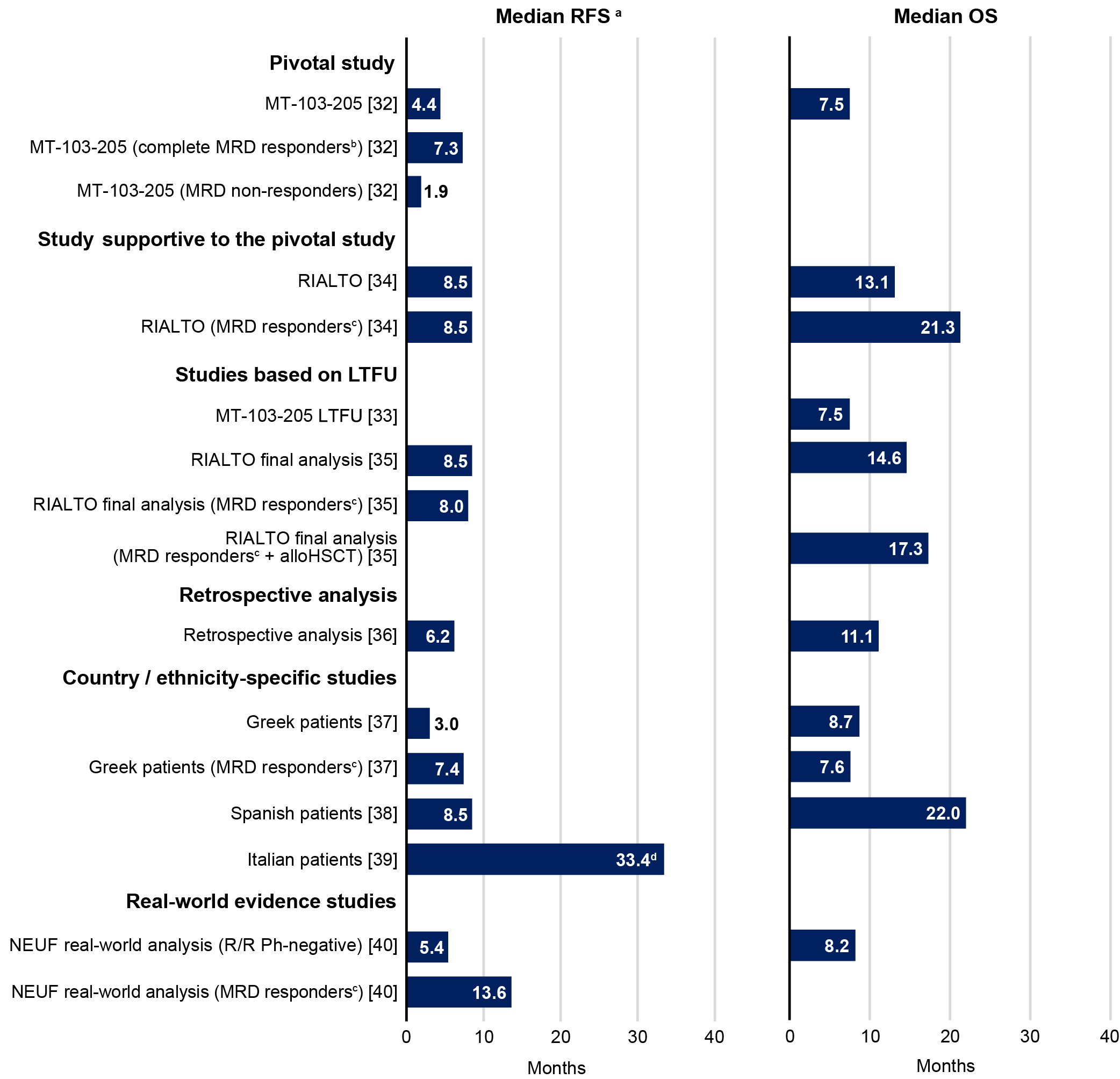

Supplement: sj-tif-5-tah-10.1177_20406207231201454 – Supplemental material for Survival outcomes in patients with relapsed/refractory or MRD-positive B-cell acute lymphoblastic leukemia treated with blinatumomab [file sj-tif-5-tah-10.1177_20406207231201454.tif]
